# The role of the body in the experience of installation art: a case study of visitors' bodily, emotional, and transformative experiences in Tomás Saraceno's “*in orbit*”

**DOI:** 10.3389/fpsyg.2023.1192689

**Published:** 2023-07-17

**Authors:** Corinna Kühnapfel, Joerg Fingerhut, Matthew Pelowski

**Affiliations:** ^1^Faculty of Psychology, Department of Cognition, Emotion, and Methods in Psychology, University of Vienna, Vienna, Austria; ^2^Berlin School of Mind and Brain, Department of Philosophy, Humboldt-Universität zu Berlin, Berlin, Germany; ^3^Vienna Cognitive Science Hub, University of Vienna, Vienna, Austria

**Keywords:** installation art, embodied cognition, aesthetic experience, interoception, body awareness, empirical aesthetics

## Abstract

Installation art, with its immersive and participatory character, has been argued to require the use and awareness of the body, which potentially constitute key parts of the artwork's experience and appreciation. Heightened body awareness is even argued to be a key to particularly profound emotional or even transformative states, which have been frequently ascribed to this genre. However, the body in the experience of installation art has rarely been empirically considered. To address this gap, we investigated the body's role in the experience of Tomás Saraceno's *in orbit* installation. Based on a list of self-report items created from a review of the theoretical literature, we—for the first time—captured (quantitatively and qualitatively): what kind of subjective bodily experiences visitors (*N* = 230) reported, how these items grouped into clusters (using network science), and how these relate to emotion, art appraisal, and transformative outcomes. Network analysis of the items determined four communities related to “interoception,” “presence,” “disturbance,” and “proprioception.” Proprioception (e.g., awareness of balance/movement/weight) turned out to be a significant determinant of art appreciation in our study, and, together with “disturbing” body experiences (feeling awkward/watched/chills), coincided with transformation. We also assessed individual differences in body awareness yet did not find that these moderate those relationships. We suggest future research on installation art based on a more unified assessment of the role of the body in embodied-enactive aesthetics and its relation to the intensity and impact of art experience in general.

## 1. Introduction

Over the last two decades, the field of empirical aesthetics has offered a wealth of behavioral and neurophysiological insights into our engagement with art. A growing number of studies have considered our reactions to art's visual properties, our emotional and cognitive responses, locations of activations in the brain related to aesthetic experience, as well as various factors which influence our reactions, such as context or individual differences (see Chatterjee and Vartanian, 2016; Pelowski and Specker, 2020 for reviews). This has led to insights into how we visually and cognitively attend to art, the neural bases for its contemplation, or when and why we might find art rewarding, arousing, or pleasurable.

At the same time, and despite this large body of research, there are still areas that have received relatively little empirical focus at both the levels of stimuli and the means of engagement: Up to now, the field has mainly assessed individual's responses to two-dimensional visual art, involving our attention to paintings or drawings, presented on screens or as prints in the laboratory (e.g., Vartanian and Skov, 2014) or, increasingly, as genuine artworks encountered in the museum (e.g., Brieber et al., 2015). However, while again important, these studies omit an important art form—installation art (Greb et al., 2017). Despite being one of the most prevalent art forms in the contemporary art world, it involves site-specific implementations or other aesthetic decisions that shape the environment itself. It has been centrally featured in many leading art shows (e.g., see the list of winners of Golden Lions in recent Venice Biennales) and may provide particularly psychologically interesting, conceptually challenging, and emotionally moving experiences (Pelowski et al., 2018). Even more, installation art specifically anticipates an aspect of engagement that, while perhaps essential both for installations and, more generally, most art experience, has itself been all but overlooked: the role and use of the body (Kühnapfel et al., 2023; Fingerhut and Spee, forthcoming).

The importance of the body for the art experience becomes apparent when considering how we engage art in free-moving, ambulatory fashions in galleries, museums, or public spaces. Theoretical literature, in turn, is abundant in assuming a constitutive role of bodily experience. It has been argued, e.g., that somatosensation and interoception are central to aesthetic experiences (Nummenmaa and Hari, 2023) or that bodily engagement is vital for meaning-making (Brinck, 2018), as well as understanding (Kai-Kee et al., 2020; Dekeyzer, 2022). Philosophers of art and art historians have gone so far as to propose that viewers' physical engagement with an artwork makes it more special, interesting, or worthwhile (Fingerhut, 2018) and is one of the aspects that makes art itself valuable as a human activity (Crowther, 1993; Budd, 1995). Similar arguments are made for our awareness of our bodies in the act of engagement (Montero, 2006; Shusterman, 2012; Jung et al., 2017; Brinck, 2018; Schino et al., 2021).^1^ These arguments also touch discourse in *Embodied, Embedded, Extended*, and *Enactive* approaches to cognition (4E Cognition, e.g., Varela et al., 1991; Newen et al., 2018), as well as phenomenological (e.g., Merleau-Ponty, 1962; Dufrenne, 1973) and pragmatism-based theory (e.g., Dewey, 1934, 1980), which suggests focusing on the situated and embodied nature when studying experience, as well as in *somaesthetics*, which recognized the body as the experiential core of perception and action and foregrounds the role of (bodily) experience in aesthetic appreciation (Shusterman, 1999). In addition, our study informs a new philosophical research project which investigates whether works of art (not limited to installation art) can be either primarily or predominantly proprioceptive in nature in that a bodily self-awareness (above, e.g., visual aspects) is required for the reception of artworks. Such artworks, among which the installation *in orbit* by Tomás Saraceno (see Methods for details) used in this study has been a frequently discussed example, could qualify as so-called “proprioceptive art” or “PropArt” (Schrenk, 2014; Kessels and Schrenk, 2022).

The role of the body becomes especially salient in installation art. Unlike two-dimensional visual art, installation art, due to its immersive and participatory character, is notably argued to evoke bodily awareness and require consideration of bodily experience (Best, 2002; Oliveira et al., 2003; Bishop, 2005). Specifically, it has been argued that active physical participation and heightened bodily presence or awareness might expand our appreciation modes and increase the emotional intensity of the installation art experience (Best, 2002; Bishop, 2005). Body awareness may also be key for eliciting potentially “profound”, self-reflective, and transformative reactions to installation art (Mills, 2009; Pelowski et al., 2018), which are also emerging as key topics in general discussions of art experience (Pelowski and Akiba, 2011; Pelowski, 2015; Fingerhut and Spee, forthcoming).

This suggests that installation art would be a particularly intriguing aspect and art form to investigate for empirical aesthetic research, considering elements of the body that might be key in appreciation. Many of those aspects, found more prominently in installation art settings, could also provide key insights into our general understanding of how we engage art or what factors might be considered in future aesthetic investigations. However, despite a few studies that have begun to consider some objectively measured body-related aspects, such as movement (e.g., Linden and Wagemans, 2021; Kühnapfel et al., 2023), types of interactions (Savaş et al., 2021; Szubielska et al., 2021), body posture (Kapoula et al., 2014), and embodiment as a component of emotions (Eskine et al., 2012; Schino et al., 2021; also see Cox and van Klaveren, 2022), to date, the body's role in art experience is only a now emerging topic in empirical aesthetics. Thus, the impact such bodily experience or engagement might have on the overall art experience remains poorly understood. Even more, the *subjective* side of bodily experience has rarely been considered, leaving the domain of self-reports largely unexplored in general and especially with installation art.

We, therefore, lack even a basic understanding of what kind of bodily experiences people might report, how they combine, and how they relate to emotional/cognitive appreciation. Furthermore, to what extent does the individuals' attention or awareness of their body modulates these responses? There is a need to assess these factors—for both installation art and potentially for a general model of our art experience and appreciation.

To address this gap, this study has two main aims. First, based on a theoretical review of the potential role of the body in art in general and specifically with installation art, we outline how the body is argued to be addressed by installation art. We also discuss what role the body might play in its appreciation or, more profound and transformative art experience outcomes. This resulted in a list of target items involving a range of subjective body awareness/experience factors that might play a role in individual reports of their art experiences.

Second, we empirically studied these bodily dimensions with a museum-based installation art piece. In this study, we also collected art emotional/cognitive/transformational experience factors and information on the visitor's interpersonal differences in body awareness. We chose the site-specific installation artwork *in orbit* by Tomás Saraceno at the K21 (Kunstsammlung NRW) in Düsseldorf, Germany. We assessed visitors' reported experiences related to their bodies when engaging with the installation to find support for the theoretical and art historical arguments suggesting that a focus on the body and bodily experiences could be central elements of art reception and appreciation. We further assessed how the body experience items grouped, employing an emerging technique for network analysis, providing information regarding the centrality, interconnections, and specific importance of items in defining the global bodily experience. The resulting scores assigned to participants were then used to consider whether/which components predict art experience regarding emotion, evaluation, and self-reflection/transformation.

In addition, to capture aspects involving the body that we did not pick up with our original assessment, we supplemented our quantitative list of factors with a qualitative approach, allowing participants to describe their body's potential role freely. Finally, we explored whether individual differences in body awareness/interoception (as assessed via MAIA-2; Mehling et al., 2018) impacted the bodily experience groups defined above or moderated any relationship between bodily experience and art appreciation.

## 2. Review: characteristics of installation art and arguments for body-related aspects

### 2.1. What is installation art?

Installation art emerged in the 1960s/70s and is—broadly conceived—a spatial-temporal and site-specific art form (i.e., works are custom-made for indoor or outdoor spaces) using a wide range of materials. This often involves a rather large presentation that visitors can enter, move around in, or at least circumambulate. As such, installation art is often not only one individual “object” but an ensemble or environment. It is, therefore, often described as “theatrical,” “environmental,” “immersive,” or “experiential” (Bishop, 2005, p. 6; also see Noë, 2000, p. 128, 131), creating conditions for bodily and interactive experiences. From its mid-century inception, installation art has become a major focus of artists and contemporary museums and received increased visitor interest (Pelowski et al., 2018, 2020; Spence, 2022). Many contemporary installation art exhibitions attract thousands of visitors to wait in long lines for often short times inside the installations (Collier, 2016; Noveck, 2017), as well record visitor numbers (e.g., 160,000 visitors saw one of the world's top-selling artists, Yayoi Kusama, “Infinity Mirror” installation; Peck, 2017, also see Neuendorf, 2018).

### 2.2. General arguments for how installation art anticipates the body

**Figure 1:**
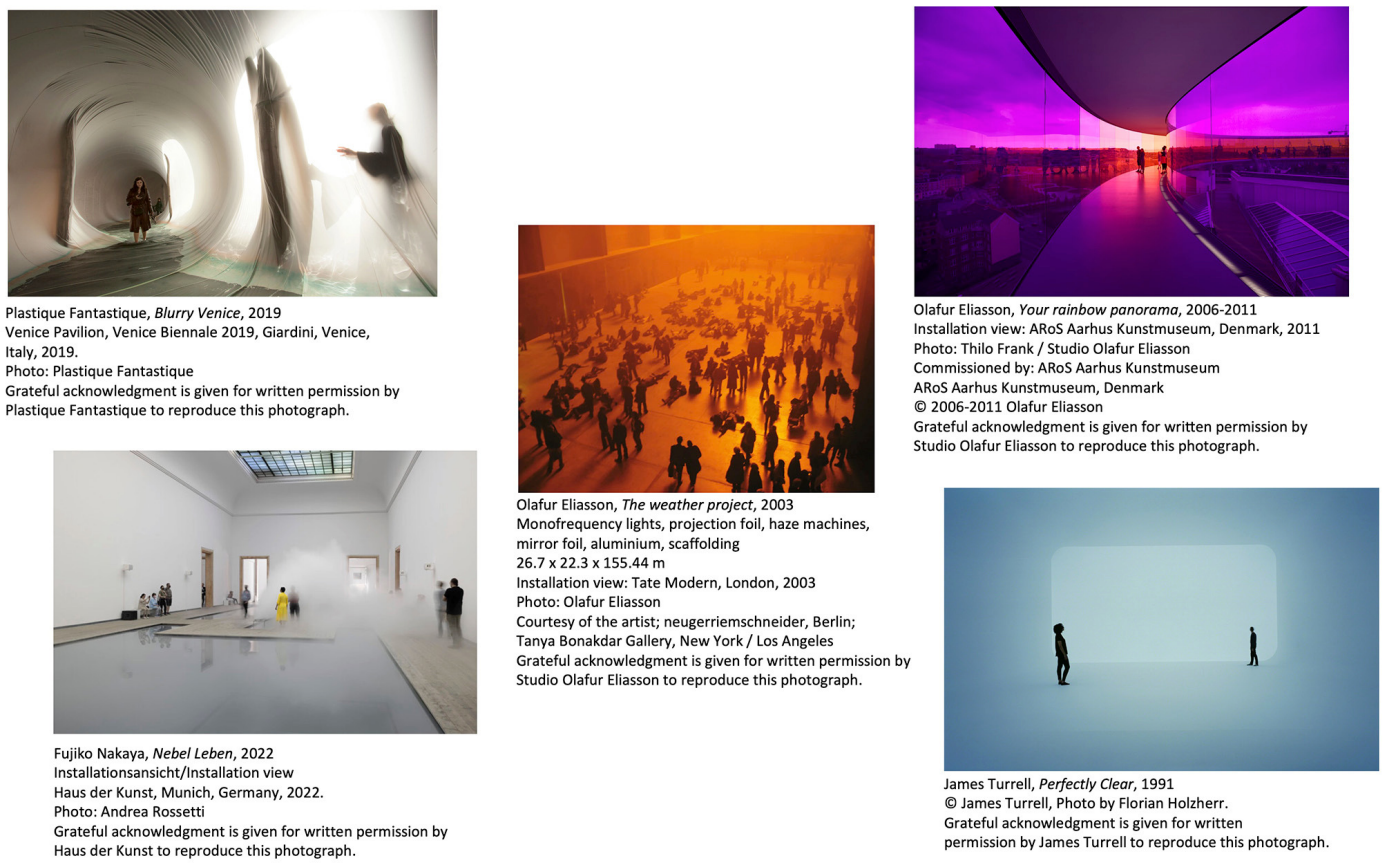
Example installation artworks that were noted to evoke and require the use of the body in various ways.

Installation art has been widely noted to evoke and require the use of the body in various capacities, above and beyond the consideration of formal properties of the art (Reiss, 1999; Best, 2002; Farkhatdinov, 2014; Caldarola, 2020; Vial Kayser and Coëllier, 2021; Kessels and Schrenk, 2022):

First, installation art generally evokes a phenomenological focus on the viewer's bodily experience and overall body awareness (Bishop, 2005; Petersen, 2015). More specifically, installation art is suggested to be often intended to increase bodily awareness and presence (Oliveira et al., 2003; Bukdahl, 2019). Such conscious experience of one's body in interaction with the installation is argued to be central to the reception of installation art (Oliveira et al., 2003; Kessels and Schrenk, 2022). For example, in response to Olafur Eliasson's large-scale installation, *The weather project* at the Turbine Hall of Tate Modern in 2003 (see Figure 1), which consisted of a large semi-circle of 100 mono-frequency laps, visitors laid down on the floor as if the installation was an actual sun, sensing the heat and brightness of it and observing themselves and others in a huge mirror hung at the ceiling (May and Eliasson, 2003; Bukdahl, 2015). In Olafur Eliason's installation artwork, *Your rainbow panorama* (ARoS, 2006–11), visitors could experience what it *feels* like to walk around inside the installation (Bukdahl, 2019; Ruiz, 2021). Yayoi Kusama's participatory mirror room installations (e.g., *Infinity Mirror Rooms* at Tate Modern, 2021–2023) immerse visitors by engulfing their bodies in patterns such that they become one with the artwork (Rosenthal, 2021). Plastique Fantastique's site-specific installation *Blurry Venice* at the Venice Biennale in 2019 gave visitors the sensation of walking on a liquid surface in a tunnel while their movements change the shape of the tunnel itself (Myers, 2019). Fujiko Nakaya's participatory installations use fog (e.g., *Nebel Leben* at Haus der Kunst, 2022) that envelops visitors allowing for a sensory connection to the fog (Sone, 2019). Bennett (2019) described their whole body feeling floating and weightless while experiencing James Turrell's Ganzfeld installation *Perfectly Clear* (1991).

Second, the participatory audience is expected to play a significant part in generating the work and its reception. The immersive and enveloping character of installation art often achieves this. Such dependence of installation art on the literal presence of the spectators has been reflected since the early 60s under the notion of “inclusion of the viewer” in art historical writings (Rebentisch, 2012, p. 248; see also Hobbs, 2001), according to which the spectator becomes “integral to the completion of the work” (Reiss, 1999, p. xii). Thus, installation art often falls under “relational aesthetics” (Bourriaud et al., 2002), where emphasis is placed on the visitor's role in the artwork's realization and reception. Bukdahl (2019) even argued that the appreciation of being participants of the artwork is part of its experience.

Last, the often-large scale and lack of perspicuity (i.e., it cannot be taken in in one glance) of installation artworks require physical activity, active exploration, and spatial movement to be able to fully explore and engage it (Noë, 2000; Bishop, 2005; Manresa, 2020). This becomes especially salient compared to paintings, which could be argued to be taken in, at least for recognition of content and visual properties, more instantaneously. As such, art experience of installation art depends upon bodily investment, which is essential for its experience. It has been argued that corporeal movement during the experience of installation art “activates [the visitors'] physical and psychic responses” rather than the art object alone (Weingarden, 2014, p. 416). Art critics also assigned new importance to this physical mode of experience: “How one saw became as relevant as what one saw” (Mondloch, 2007, p. 23).

### 2.3. The role of the body for art experience (of installation art)

In turn, on the basis that the body is part of its reception, there are multiple arguments that the body plays a key role in the nature of the experience and the appreciation and evaluation of the art.

Both components are intertwined. Bodily engagement and experience might be considered important elements of aesthetic interaction with artworks that “*de facto* contribute to our appreciation” (Fingerhut, 2018, p. 84). Best (2002) suggests a constitutive role of the body for the appreciation of installation art in that the body expands the modes of apprehension in addition to more common ways such as vision and the intellect. Furthermore, another important characteristic of installation art, participation, has been argued to be vital for appreciation: “the viewer has to be physically present in the work or a performance of it and has to behave in the prescribed manner while there, so as to enhance his or her appreciation of it” (Novitz, 2001, p. 154). In terms of the participatory role of the visitor discussed above, as opposed to a passive “recipient,” in installation art, the participatory visitor is invited to contemplate their role in the installation, which might lead to a more self-aware and reflective state, and thus heightened art experience (Rebentisch, 2012).^2^

Regarding the role of active movement in appreciation for the experience of architecture, which parallels installation art, it has been argued that the sequence of experiences consequent on movement through a building may be essential to its effect on the viewer. “The feeling of our bodies moving through a building, reaching out and touching its surfaces, listening to its echoes, and so on is a crucial part of appreciation in its own right” (Robinson, 2012, p. 338). Shifting our awareness to our body or general capacity for body awareness could also magnify bodily sensations, which in turn could heighten our aesthetic experience (Montero, 2006; Lanzoni, 2009; Shusterman, 2012). Oliveira et al. (2003) argued that the degree to which our sensory faculties are stimulated in the experience of installation art is linked to the impact that an experience can have on us.

Again, to give an example with architecture, Shusterman (2012) suggested that “If the appreciation of architecture is so strongly linked to somatic experiences [such as proprioception], then heightening somatic consciousness could improve our architectural experience” (p. 14; see Montero, 2006 for a similar argument regarding the enhanced experience of watching and performing dance though shifting awareness to the body). These arguments further amplify that bodily awareness and experience may play an aesthetically relevant role in the receiver's experience with the addition that more bodily awareness/experiences might heighten art experience, especially of installation art.

### 2.4. The body and profound or transformative experience

Beyond the proposed role of the body for the appreciation of the artwork, installation art has specifically been argued to cause profound and complex emotional involvements (Bishop, 2005; Vial Kayser and Coëllier, 2021) or even to have transformative potential (Weingarden, 2014, 2015; Sherman and Morrissey, 2017; Yoshitake et al., 2017; Vial Kayser and Coëllier, 2021).

To name a few examples, Carsten Höller said about his participatory installations that “they offer the possibility of unique inner experiences that can be used for the exploration of the self” (Bukdahl, 2015, p. 179, Interview with Carsten Höller by Vincent Honor, *op. cit*.). The immersive Ganzfeld environments created by artist James Turrell caused visitors' perception to “become the object of reflection, and led some to a deeper understanding of themselves and their relationship to the external environment, deepening their conception of themselves as embodied beings” (Sherman and Morrissey, 2017, p. 6). Similarly, in discussing his works, Eliasson (2019, p. 19) suggests that increased self-reflection through the awareness of the body and processes of perception “leads to a more reflective attitude toward the world outside the artwork.” By what means people have profound, life-changing experiences within installation art has received increased interest (Pelowski and Akiba, 2011; Weingarden, 2014, 2015; Pelowski et al., 2018; Sadia, 2021).

In fact, a factor that potentially makes the encounters resonant may be the heightened physicality itself. The basis for heightened physicality is the temporal, spatial, and immersive scale of installation artworks, which has been argued to allow reflection on how the spectator feels inside it (Bishop, 2005) or in their presence: “the works make us reflect on how we *feel*, […], in their presence” (Noë, 2000, p. 131), which in turn facilitate self-awareness and deeper consideration (Pelowski et al., 2018). Body awareness, in general, arguably plays a central role in all subjective experiences (Berlyne, 1971; Djebbara et al., 2019) in that it heightens our awareness of what is around us and ourselves, thereby making us aware of what we are feeling (Brinck, 2018).

One prompt for a switch to an inward reflective mode has been specifically argued to be aware of one's body. Theoretical writings have drawn the notion of the body/self as a “modality of reflexivity” (Jones, 2000, p. 335), and awareness of the body has been argued to be one of the crucial elements in the process of also becoming aware of oneself (Berlucchi and Aglioti, 2010; Pelowski, 2011; Jelić and Staničić, 2021). In a museum study involving Mark Rothko paintings, which covered all gallery walls in a particularly immersive manner, Pelowski (2015) showed that the body could play an essential role in inducing more self-reflective responses. The author reports that contextual sensations (e.g., feeling watched, hearing one's footsteps, getting chills from the air conditioning system) may shift attention to one's body and current actions, especially when facing particularly engrossing or challenging art. This awareness may induce meta-cognitive reflection about one's experience (Pelowski et al., 2017). Altogether, bodily awareness might function as a crucial trigger for making people more receptive and reflective.

To take the discussion a step further, besides the capacity for general increased body awareness through the spatial character of installation art, installation art often evokes direct and powerful corporeal relations by, e.g., intervening on our balance or posture (Bishop, 2005). By foregrounding certain spatial features or leading/disturbing the visitors' movement through them, installations serve to force an awareness toward one's body and responses which were taken for granted in non-art spaces, and “individuals may even come to deeper appreciation by juxtaposing visual and proprioceptive sensations (awareness of being in an [installation])” (Pelowski et al., 2018). Such a specific interaction of interoception and exteroception could also be a hallmark of aesthetic emotions that can lead to more reflective modes of engagement (Fingerhut and Spee, forthcoming).

In architecture, switching visitors' attention to the body has been successfully employed by architects throughout history to immerse a perceiver into the spatial situation. In moving through space, the body schema is responsible for the continuous tracking of bodily states and positions as we move by integrating interoceptive, proprioceptive, and exteroceptive information and having an awareness of ourselves as embodied beings (Jelić et al., 2016; Jelić and Staničić, 2021). Rupture of this habitual body schema, such as when engaging architecture (or installation art), then activates an attentional switch that allows “the visitor to consciously experience the […] setting and oneself as an experiencing and bodily subject” (p. 7). For example, the architectural design of Carlo Scarpa's stairs at the Brion Cemetery in San Vito d'Altivole, Italy, is argued to afford each step to be performed with specifically one of but not either the left or right foot, which “ruptures” one's usual stair-walking behavior to allocate an attentional switch to the conscious experience of one's body (Jelić et al., 2016). Thus, strong bodily experiences might be crucial triggers to make ourselves more receptive and make the whole experience more profound and meaningful (also see Robinson, 2012, 2021).

### 2.5. Previous empirical research on installation art

In sum, the above review provides a large number of items that could be related to art experiences with installations and with art, in general, relating to aspects from the basic requirement for engagement with our bodies, to modes of interaction/participation, to various ways of being aware of our bodies and movements, as well as to corresponding or interrelated emotions and cognitive or other responses. Each of these could be treated as potential hypotheses that might individually, or in combination, play a key role in individuals' reported art experience (see also Table 1, and further discussion in the methods).

**Table 1:** Twenty-nine self-report body-items (rated on 7-point Likert scales; 1 = Not at all, 7 = A lot).

**#**	**Body-item *(I felt…)***	**Motivations for the choice of body-items related to (a) experiences of installation art in general, (b) Tomas Saraceno's *in orbit*, or (c) transformative art experience outcomes**
1	A change in body temperature	Sensing a change in body temperature can be one of the various kinds of bodily experiences evoked in the experience of installation art. For example, anecdotal reports of Olafur Eliasson's large-scale installation *The weather project* at the Turbine Hall of Tate Modern in 2003 (see Figure 1) show that sensing the heat of the light of the installation was part of its experience and might have changed visitors' body temperature.
2	Sweat	This item is possibly related to #1. Anecdotal reports of Saraceno's *in orbit* note a sensation of sweaty palms (e.g., Putnam, 2014).
3	A sense of being watched	Feeling watched/triggering a feeling of observation can occur in real art museum/gallery contexts and has been argued to be an external trigger inducing focus back on oneself (i.e., self-awareness) and reflection on art experience Pelowski, 2011. As *in orbit* is an example of museum-based art where visitors are present, we might expect feelings of being watched.
4	Chills/goosebumps	Chills are common bodily responses to art (e.g., Silvia and Nusbaum, 2011), and are especially inhibited by discrepancies in processing experience (for review, see Pelowski and Akiba, 2011).
5	A sense of vertigo	Some installation artworks have been noted to play with feelings of vertigo. For example, Kim Levin has described the experience of walking through Lucas Samara's *Mirror Room* installation at Pace Gallery, New York as vertiginous Levin and Samaras, 1975. Furthermore, anecdotal reports of Saraceno's *in orbit* note vertigo (e.g., Putnam, 2014).
6	Grounded	Installation art has been noted to evoke a sense of being fully embodied in which one of consciousness is a present moment/situation (e.g., being “grounded in an experience of the body”, Bishop, 2005, p. 107; also see Bennett, 2019).
7	My body shaking	Installation art often intervenes with one's posture and balance, for example uneven or unstable grounds. This can also be caused by the movement of other visitors in Saraceno's *in orbit* (e.g., I felt “the vibrations which the other visitors put into the net,” Boehling, 2016, as well as the height causing instability leading to body shaking)
8	As if I would fall over any moment	Installations that play with alternative grounds like *in orbit*, might give the visitors a feeling as if they would fall. Anecdotal reports of *in orbit* describe what might lead to a sensation of falling: “If it is entered by more than one person, the installation is set in motion” (Chin, 2013); “People walk, lie or stand unsteadily within it.” (Wattolik, 2019); “Visitors find themselves confronted with the issues of flying, falling and floating” (Chin, 2013).
9	Revived	Bodily arousal might be connected to revived experiences (“revived” was frequently reported in a study using installation artworks by Olafur Eliasson in Pelowski et al., 2018). The stimulating experience created through the height in *in orbit* might evoke feeling revived (e.g., Putnam, 2014).
10	Exhausted	Installation art requires active movement and physical effort to take in and explore their often large scale (Bishop, 2005). Furthermore, *in orbit* requires movement and the use of muscles to stabilize one's body in the net (Putnam, 2014; Kessels and Schrenk, 2022), which might feel exhaustive after some time.
11	Awkward	Experiences with installation art have been related to awkwardness (and discomfort) (e.g., Sierra's work in Bishop, 2005). To give an example with Saraceno's *in orbit*, visitors' movements have been described as “awkward” (Putnam, 2014).
12	Weightlessness	Bennett (2019) described that her whole body responded with a sensation of floating and feeling weightless while experiencing James Turrell's Ganzfeld installation *Perfectly Clear* (1991). Furthermore, experiences of “weightlessness” have anecdotally been part of Saraceno's *in orbit* (Frank, 2013).
13	Disorientated	Some installation artworks have been noted to play with feelings of disorientation (Bishop, 2005). For example, Kim Levin has described the experience of walking through Lucas Samara's *Mirror Room* installation at Pace Gallery, New York as disorienting (Levin and Samaras, 1975),
14	Immersed	Installation art is often intended to immerse the viewer (Bishop, 2005). Furthermore, *in orbit* was described as immersive (Putnam, 2014).
15	As if I were part of the artwork	Viewer participation is an important characteristic of installation art (Reiss, 1999; Hobbs, 2001; Rebentisch, 2012). Visitors play an important role of the visitor in the realization and reception of the artwork. For example, Yayoi Kusama's participatory mirror room installations (e.g., *Infinity Mirror Rooms* at Tate Modern, 2021–2023) immerse visitors by engulfing their bodies in patterns such that they become part of the artwork (Rosenthal, 2021). The role of the participatory audience in *in orbit* especially becomes apparent in this statement on the installation by artist Tomás Saraceno: “If there is no person in the work, you don't see the work. It is invisible.” (Trailer: Tomás Saraceno—in orbit, 2013).
16	Unable to move	Being unable to move has been noted to be part of transformative art experience (Pelowski, 2011), and associated with bodily expressions of wonder (Fingerhut and Prinz, 2018); also see “Stopping for knowledge” hypothesis; (Sarasso et al., 2020).
17	My movement/actions	Often movement is required to fully explore and engage installation art (Noë, 2000; Bishop, 2005). Olafur Eliasson described his installations so that the visitor can experience “what it feels like to walk around inside my installation” (Bukdahl, 2019, p. 64). To give another example, Plastique Fantastique's site-specific installation *Blurry Venice* at the Venice Biennale in 2019 gave visitors the sensation of walking on a liquid surface in a tunnel, while their movements change the shape of the tunnel itself (Myers, 2019). Awareness of the way of movement (e.g., walking) has also been noted in anecdotal reports of *in orbit* (e.g., Putnam, 2014).
18	My body	General body awareness has been noted to be part of the reception of installation art (e.g., Oliveira et al., 2003; Kessels and Schrenk, 2022), and has received high ratings from participants in an empirical study with installation art by Olafur Eliasson (Pelowski et al., 2018).
19	My existence in the world	Installation art has been suggested to often be intended to increase the presence and awareness of the here and now in the world (Oliveira et al., 2003; Bukdahl, 2019).
20	The space/environment	Reiss (1999) and Bishop (2005) argued that with installation art, visitors not only become aware of the body but also the environment/space created by the installation/in which the installation is set up experiences (e.g., forms, proportions of the gallery, how objects are installed in space).
21	My posture	Several studies showed that artworks can have a measurable impact on body posture (e.g., see Kapoula et al., 2014 for a study with Richard Serra's *Promenade*), while the subjective experience of feeling one's posture has not yet been assessed.
22	My balance	Installation art often evokes direct and powerful corporeal relations by intervening with our balance or posture, e.g., via the use of uneven floor; Bishop (2005). Anecdotally, balance is an aspect of bodily experience noted by visitors of *in orbit* (e.g., Boehling, 2016; Hanz, 2019).
23	The weight of my body	Awareness of one's body weight has been noted in connection with experiences of installation art (Bishop, 2005).
24	Movements of other visitors	Environmental “triggers” such as sensing the movement of others may play a relevant role in art experience in terms of inducing meta-cognitive reflection, insight, and positive assessment (Pelowski, 2012). Noting the movement of others might be especially part of the experience of *in orbit* because “The net vibrates as other participants move though it” (Putnam, 2014).
25	My core muscles	Sensing one's heartbeat has been argued to be part of so-called proprioceptive art or PropArt, which installation art often applies to, and noted to be part of the experience of *in orbit* (Kessels and Schrenk, 2022): “the installation is not only experienced visually and haptically, but also the perception of the movement of one's own body in space and the tension of the muscles in the limbs and torso, as well as possible effects on the heartbeat, breathing, and general stress level are an Integral part of the reception of the artwork”^3^ (p. 3).
26	My heartbeat	Sensing one's heartbeat has been argued to be part of so-called proprioceptive art (Kessels and Schrenk, 2022), which installation art often applies to. It has been noted in anecdotal reports on the experience of *in orbit* (e.g., Putnam, 2014). Furthermore, see item #26.
27	My breathing	See item #26. Awareness of one's breathing has been noted to be part of the experience of *in orbit* (e.g., Putnam, 2014).
28	Gravity	Sensing one's gravity has been a part of the experience with installation art. For example, Bennett (2019) reported on the experience of James Turrell's Ganzfeld installation *Perfectly Clear*: “My sense of distance, of gravity, of my physical placement in the room, all dissipate. I float in light” (p. 3). Sensing one's own weight in relation to the ground has also been reported in relation to *in orbit* (Hanz, 2019).
29	Absorbed	Installation art is often intended to absorb the viewer (Bishop, 2005). Note, this item was excluded from network analysis because many participants did not understand the term absorbed (“absorbiert” in German) (see Results).

Looking at past studies on installations, however, only some of the above aspects have been considered. A few studies have started investigating other relevant parts of the engagement and experience of installation art besides the role of subjective bodily experience and awareness. For example, Gulhan et al. (2021) found that gaze behavior inside an installation artwork vs. a virtual reality reproduction did not significantly differ. Another study found that a contemporary critical art exhibition, including installation art along with other mediums, received higher aesthetic appreciation when experienced in a gallery as opposed to a laboratory context (Szubielska et al., 2021), as well as was better understood when a curatorial description was available (Szubielska and Imbir, 2021).

Other studies graphically showed that the spatial arrangement of a sculpture affected visitors' movements and physiological responses during a gallery visit via maps of movement paths and where on the paths physiological responses (heart rate and skin conductance) were stronger (also see Tröndle and Tschacher, 2012; Tröndle et al., 2014), or assessed more complex emotional/cognitive experiences in combination with viewing behavior (Pelowski et al., 2018). A series of studies started to specifically compare art experience between interactive art installations and non-interactive versions of them, while the authors agree that more research is needed because results on this comparison remain mixed (e.g., Jacucci et al., 2009; Vi et al., 2017; Savaş et al., 2021; Szubielska and Imbir, 2022).

Regarding the body, one study assessed how installation art could have a measurable, objective impact *on* the body. Kapoula et al. (2014) showed that Richard Serra's *Promenade* installation improved visitors' balance after walking around and alongside the artwork's laterally tilted monumental elements that play with depth and verticality (see also Nather et al., 2010; Kapoula et al., 2014; Vernet et al., 2018 for examples of how representation of movements or depth in visual art has been shown to modulate posture control). Kapoula et al.'s (2014) study did suggest that spatial properties of installation art can have an *objective*, measurable impact on the body, and the importance of ecologically valid testing.

However, none of the above studies considered viewers' *subjective* experience of the body and how this relates to installation art experience and evaluation. We (Kühnapfel et al., 2023) previously used self-reported bodily items and found that on average participants were generally aware of their body and movement in front of a painting, their distance from and approach to it and that the room/space had influenced the way they encountered the painting while also showing variability in these responses. To our knowledge, only one study with installation art has included assessments of subjective body awareness in a self-report questionnaire. Pelowski et al. (2020) recorded individual's experiences with three different installation artworks to assess whether they matched what the artists intended. After engaging each installation artwork, participants reported their emotional and phenomenal experience based on a list of 37 self-report items, each rated on an 8-point Likert scale. One item out of these was “awareness of the body”. The mean ratings on “awareness of the body” were among the highest noted ratings in the reports on two of the installation artworks (i.e., second highest rating out of the 37 ratings after “feeling stimulated” for artwork two, and fourth highest out of the 37 ratings after feeling “a sense of confusion,” “stimulated,” and “absorbed” for artwork three). This study suggested that body awareness could be one experience dimension that is rated as considerably high, compared to other emotional and phenomenal experience factors. However, this study did assess only this single item on “awareness of one's body” and did not relate it to other experience factors, missing to capture or address important nuances of bodily experience and interactions, as well as related to other aspects of art experience.

### 2.6. Trait awareness of the body and relation to body/art experience

The review so far suggests that relatively more awareness of one's body also modulates our bodily experiences, which in turn shapes the experience of an artwork. One further question should be *who* becomes aware of these bodily experiences. Interpersonal differences in awareness of one's body might translate to different impacts of body awareness or even general responses when engaging in art.

However, to date, no study in empirical aesthetics using visual arts has assessed the role of individual differences in body awareness in art experience. Regarding the experience of dance, researchers found that the ability to accurately detect interoceptive signals (i.e., to count one's heartbeat in given time windows accurately) modulated the relationship between physiological sensitivity (in terms of Galvanic Skin Response) to dance stimuli varying in expressivity and rated expressivity of the stimuli (Christensen et al., 2021; also see Christensen et al., 2018).

## 3. Methods

The present study explored participants' subjective experiences of their body and potential body-related factors, which had been mentioned in the review, and their impact on art experience and evaluation.

### 3.1. Participants

We involved a final sample of 235 participants (142 female, 89 male, 2 other, 2 preferred not to answer, *M*_*age*_ = 33.82, *SD* = 14.36, range = 18–72 years). The researchers tried to invite as many museum visitors as possible to participate in the study in the time the museum gave. These individuals constituted a convenience sample of museum visitors. Participants were eligible to participate in the study if they were older than 18 and proficient in English or German. Participants received a museum visit voucher (worth 12 €) as participation compensation. The final sample was derived from an initial collection of 236, with one individual not meeting the participant criteria (under age 18). The study was conducted according to the Declaration of Helsinki and the University of Vienna Ethics Committee. A signed informed consent was completed before participation.

### 3.2. Stimuli

**Figure 2:**
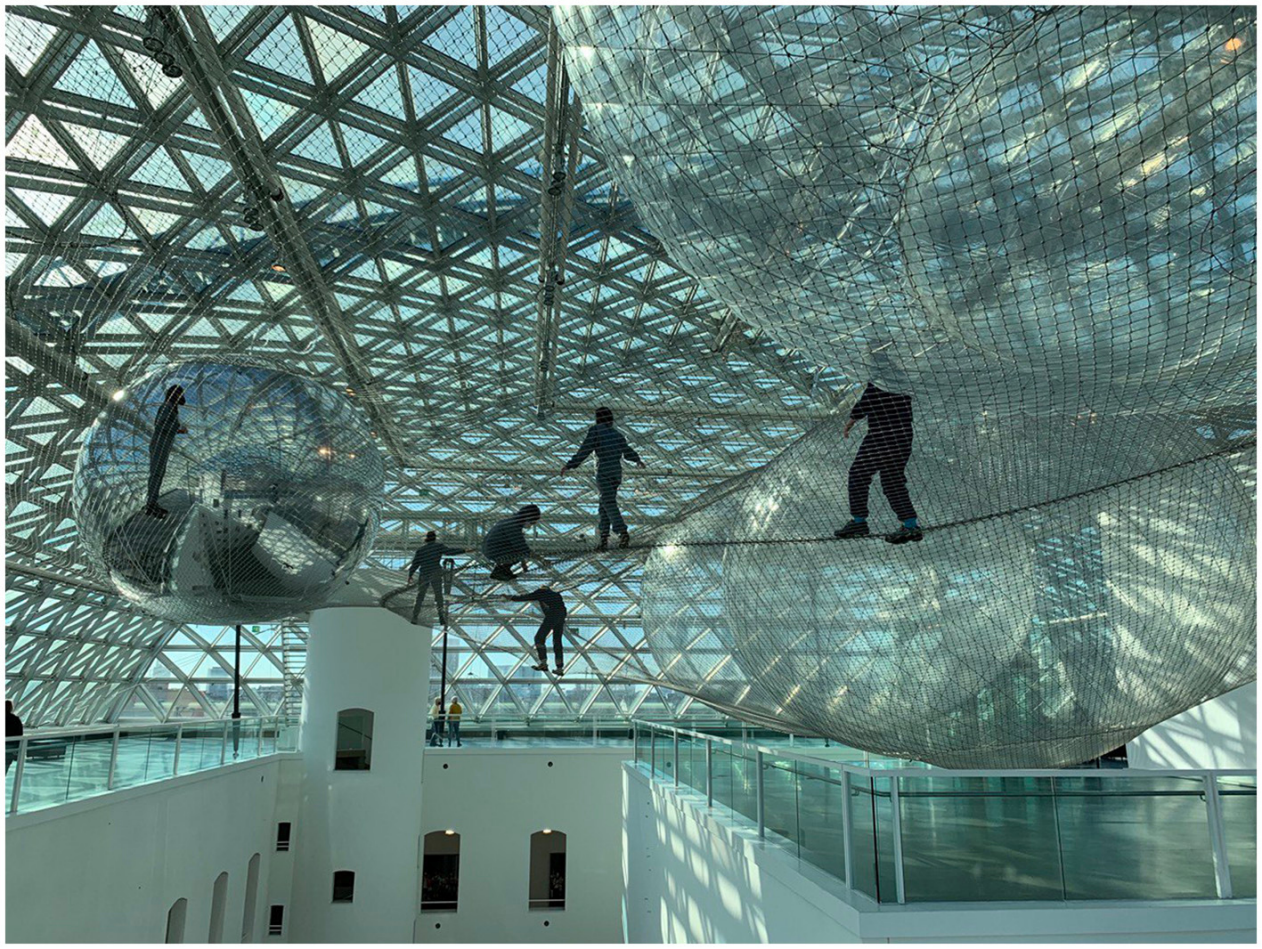
Tomás Saraceno—*in orbit*, installation view K21, Düsseldorf, Germany. Tomás Saraceno—in orbit, 2013,-ongoing, installation view K21, ©Tomás Saraceno. Grateful acknowledgment is given for written permission by Studio Tomás Saraceno to reproduce this photograph.

The stimulus was the installation artwork *in orbit* by Tomás Saraceno (^*^1973), exhibited at Kunstsammlung Nordrhein-Westphalen (K21) in Düsseldorf, Germany, curated by Marion Ackermann and Susanne Meyer-Bser. The installation (see Figure 2) is composed of a 2,500 m^2^ (880 m^2^ walkable area) three-layered net made from 3 mm-diameter stainless-steel cable spanned horizontally at a considerable height of 25 meters over the atrium of the K21, upon which visitors could enter and explore the different interconnected layers of the net or sit/lay while suspended in space. The net's three levels were spread apart from each other by five air-inflated PVC (3.5–8 m ø) shells in the form of spheres made of PVC. The installation was part of a larger project called “Cloud Cities,” which addresses the ideas of flying cities or living clouds (Ackermann et al., 2011). Like a spider/web, visitors can feel the movements of others in the net, echoing Saraceno's artistic practice and research on hybrid and interspecies forms of communication (Kunstsammlung NRW, 2013). Visitors had to bring their own sturdy footwear (e.g., hiking boots, no sneakers) or borrow suitable footwear from the museum. The museum also provided overalls to wear on top of one's clothes as protection. Visitors were not allowed to bring any objects into the installation, had to take off jewelry or watches, and wear an FFP2 mask (due to COVID-19 protocols).

This installation artwork was chosen because it matched several of the above-discussed aspects, such as audience participation, bodily engagement, and awareness of one's body in general and various bodily experiences, which are argued to be integral to the reception of this installation, as well as its connection to profound/self-reflective experiences. The installation *in orbit* is participatory in that visitors must enter it to experience it fully (Putnam, 2014,^4^). The role of the participatory audience in completing the work especially becomes apparent in this statement on the installation by artist Tomás Saraceno: “If there is no person in the work, you don't see the work. It is invisible.” (Kunstsammlung NRW, 2013).

According to anecdotal reports, visitors routinely report experiences related to the body in terms of proprioceptive (e.g., awareness of one's movement or sensing tension of one's muscles) and interoceptive sensations (e.g., awareness of one's breathing or heart pounding) (Putnam, 2014; Hanz, 2019; Wattolik, 2019; Kessels and Schrenk, 2022; Spence, 2022). *in orbit* also provides a perfect example of how bodily experience is triggered to make the art encounter resonant (Jelić et al., 2016): as visitors enter the participatory installation, their bodily experience is stimulated or even ruptured in terms of altering visitors' posture, balance, or perception of body weight (Frank, 2013; Trailer: Tomás Saraceno—In orbit, 2013).

Engagement with the installation also offers profound/self-reflective experiences as reported in anecdotal reports and interviews with the artist. In the exhibition trailer, the artist, Tomás Saraceno, describes his intentions: “I want to provoke feelings, make people more sensitive to the installation, other people, or even the weather. And who knows, maybe this sensitivity will lead to more peace. […] I am interested in how this installation generates new ideas” (Trailer: Tomás Saraceno—In Orbit, 2013). The text about the installation on the museum website states the following intentions: “*In orbit* is a methodological attempt to achieve an ethical sensitivity, becoming aware of the many phenomena that shape our possibilities of being-in-the-world and our role in it, making them perceivable through a unique synesthetic experience.” Visitors have anecdotally reported such experiences of self-reflection and perspective change (e.g., Chin, 2013,^5^; Divisare, 2013,^6^), as well as other thought processes, argued to be triggered by the environment created by the artist (Prinsi, 2013).

### 3.3. Procedure

Data were collected over 10 days from the 9th to the 19th of March 2022 (11:00 a.m. to 5:30 p.m.). This ending time allowed us to stop collection before sunset so that the presence of daylight and/or artificial lights was relatively consistent for all participants. A maximum of seven people were allowed to enter the installation for approximately 10 min for safety reasons.

We employed a collection design whereby participants were stopped by a researcher immediately *after* they had exited the installation and asked to participate. This allowed us to assess the experiences spontaneously had by participants without prior priming or fore-knowledge that individuals would be participating in a study.

Participants were asked to read and sign the informed consent if they agreed. The survey was administered via the online commercial survey tool Qualtrics (Qualtrics Int., Seattle, WA, USA). It could be accessed via a QR code, allowing participants to answer questions on their own smartphones or via tablet computers provided by the researcher. Participants could take the survey in German or English. The survey lasted about 20 min. After the experiment, participants were debriefed about the purpose of the study and handed the museum ticket voucher as compensation.

### 3.4. Measures

#### 3.4.1. Bodily experience

The main aim of the survey was to collect participant reports regarding a range of reactions tied to awareness of the body (or, alternatively, to assess whether and to what extent such factors were noted by participants as part of their experience). This was accomplished in two ways—a survey (quantitative) based on the above review of theoretical and anecdotal arguments and an open-ended free answer (qualitative assessment).

##### 3.4.1.1. Quantitative survey

Participants reported their subjective feelings or awareness of body-items using a list of 29 factors created by our research team, which are summarized in Table 1. The items were based on the above review regarding the possible way the body might be involved in the experience of installation art and with the further aim of later assessing the items' importance and interrelation using network science. The items captured the participation/feeling part of the artwork, inner (e.g., heartbeat) or outer (e.g., chills) bodily sensations, awareness of the space/visitors around one and one's existence in/relation to it (e.g., groundedness, immersion). We also included some contextual sensations that have been argued to trigger awareness of one's body (e.g., feeling the movements of others or a sense of being watched) (Pelowski et al., 2014), as well as rather negatively balanced experience aspects that could components of art experience in real-world settings (e.g., feeling exhausted, or disoriented) (Pelowski and Akiba, 2011; Pelowski et al., 2014).

Items were introduced with the instruction, “Please think about your art experience. While engaging with the art, I felt…” (e.g., “my body,” “my breathing,” and “my heartbeat”), which participants rated on continuous 7-point Likert-type scales ranging from 1 = “Not at all” to 7 = “A lot.” The 29 items were displayed as two lists, to split up the body item list into two pages. The first list included the first 17 items of Table 1, and the second list included the last 12 items. Item order was randomized within each list. To assess whether participants enjoyed their bodily experience, we also asked the question, “How much did you enjoy your overall bodily experience?” on a 7-point Likert-type scale (1 = “Not at all” to 7 = “A lot”).

##### 3.4.1.2. Qualitative assessment

We also invited participants to reflect upon and report experiences related to their body (if any) via filling out an open text box. This assessment was placed before the body-item list to allow us to assess the spontaneous subjective reports independent of possible priming effects from our scale-based questions. We believe that it is important that some associative, qualitative analysis should complement the quantitative survey/network model. This provides us with more vivid, subjective reports that can be used to interpret the resulting network communities (i.e., how the experience in each community might have felt) and a bottom-up way to discover factors not anticipated in our review of relevant body-items.^7^ The qualitative data analysis was performed before the network analysis such that the researchers were not biased by it.

#### 3.4.2. Art experience

We included additional questions to assess other factors relating to art evaluation or notable emotional/transformative responses to the art. To measure art evaluation, we asked how much participants “liked” the installation, as well as how “meaningful” and how “interesting” they found it. We focused on these three appraisals as they were consistently noted in the review above about bodily experience and are part of common in empirical aesthetics assessments (e.g., Brieber et al., 2015; Tinio and Gartus, 2018; Specker et al., 2021; Kühnapfel et al., 2023), but also assessed a series of other potentially relevant appraisal factors that are reported with the descriptives and correlations to each other in Supplementary Table 2 but were not focus for the present analysis. One of those items was whether participants assessed the installation as “good” art, which is underrepresented in empirical aesthetics (Fingerhut and Prinz, 2018) and will be addressed in future studies.

To capture potential self-reflective and perspective-changing experiences, we asked for “self-reflection” and “gaining a new perspective,” which are associated with a transformative/schema change outcome (Pelowski et al., 2017, also see Pelowski and Akiba, 2011). In addition, as described in the stimuli section, perspective change and self-reflection were experiences noted explicitly by the artist and anecdotal reports. Finally, we also asked whether participants experienced “a transformation” to directly capture the potential transformative character of installation art in general in a subjective way (Weingarden, 2014, 2015; Sherman and Morrissey, 2017; Yoshitake et al., 2017; Vial Kayser and Coëllier, 2021). All items were rated on 7-point Likert scales ranging from 1 = “Not at all” to 7 = “A lot.”

In addition, we included two specific emotional terms regarding how much participants experienced “awe” and “wonder.” These were selected because they are often noted as salient feelings regarding particularly profound aesthetic or art experiences (Fingerhut, 2018; Fingerhut and Prinz, 2018). In addition, awe and wonder have been argued to play a role in stimulating new ways of thinking and understanding (Pelowski et al., 2017), schema/knowledge change (Keltner and Haidt, 2003; Shiota et al., 2007), personal meaning, transformation, and profound experience (Schneider, 2009; Cohen et al., 2010; Nusbaum and Silvia, 2014), and are connected to self-awareness (Yaden et al., 2017). These two terms were also mentioned anecdotally in conjunction with *in orbit* in online articles (e.g., Jones, 2013; Boehling, 2016).^8^

#### 3.4.3. Individual differences in trait body awareness

We employed the Multidimensional Assessment of Interoceptive Awareness Version 2 (MAIA-2; Mehling et al., 2018) to assess individual differences in interoceptive bodily awareness. The MAIA-2 is a validated and established state-trait questionnaire to measure multiple dimensions of interoception by self-report. It consists of 37 statements, which participants rate on a 6-point Likert scale ranging from “Never” to “Always.” An overall score is calculated by summing and averaging all items, which was also the main assessment for the present study. This total ranges from 0 to 5, with higher scores reflecting greater interoceptive sensitivity. The original MAIA-2 questionnaire was used in the English version of the survey, and the German version of the MAIA-2 was used in the German survey (for the validated German version see Bornemann et al., 2015; Eggart et al., 2021).

In addition, the battery can also be divided into eight subscales: (1) “Noticing” changes in one's body when being uncomfortable (e.g., *I notice when I am uncomfortable in my body*), (2) “Not-Distracting,” or the tendency to ignore or distract oneself from sensations of pain or discomfort (e.g., *I distract myself from sensations of discomfort*), (3) “Not-Worrying,” emotional distress or worry with sensations of pain or discomfort (e.g., *When I am in discomfort or pain I can't get it out of my mind*), (4) “Attention Regulation,” ability to remain focused on the body even when distracted (e.g., *I can return awareness to my body if I am distracted*), (5) “Emotional Awareness,” ability to notice changes in one's body when feeling a certain way (e.g., *I notice that my breathing becomes free and easy when I feel comfortable)*, (6) “Self-Regulation,” one's ability to use focus on one's body to reduce stress or tension (e.g., *I can use my breath to reduce tension*), (7) “Body Listening,” using one's body to learn about one's emotional state (*I listen to my body to inform me*), and (8) “Trust,” seeing one's body as a safe place and trusting one's sensations (*I trust my body sensations*).

#### 3.4.4. Demographics, familiarity, and social experience

Finally, we asked several questions to collect more contextual factors that might have influenced experiences or reports. These included: familiarity, assessed by asking whether participants had experienced the installation before (excluding the time just before the survey) and, if yes, how many times. We also asked participants whether they experienced the installation alone or with others (e.g., friends, family, colleagues), following studies suggesting that experiencing art together vs. alone can influence the art experience (Pelowski et al., 2014 for review). Art engagement, in terms of frequency of museum visits (pre–COVID-19 crisis), art education, fear of height, art preference, and demographical data (age and gender), was also recorded.

## 4. Results

An overview of the participant demographics and other characteristics can be seen in Supplementary Table 1. Overall, our participant sample was generally interested in art (*M* = 6.23, *SD* = 1.63; on a 7-point scale). However, 78.7% responded that they had no prior art education/knowledge. For 83.4% of the sample, it was the first time they had encountered the installation, while the remaining 11.1% had visited an average of 1.5 times before (range = 1–3 previous visits). Most participants (83.4%) went to the installation with friends/family/colleagues. The average score on the fear of heights rating was on the positive side of the scale (*M* = 4.39, *SD* = 2.19; out of 7 points), with just over half of the participants (52%) scoring on the positive side (ratings 5, 6, 7) but with 45% suggesting relatively high agreement (6–7 points).^9^

Our sample scored moderately high in the overall MAIA-2 score, with the highest distinctiveness in the “Emotional Awareness” subscale, which indicates the ability to notice changes in one's body when feeling a certain way, and the lowest in the “Not-Distracting” subscale, which indicates the tendency to ignore or distract oneself from sensations of pain or discomfort (see Supplementary Table 1). In the current sample, Cronbach's α indices of internal consistency reliability of the MAIA-2 was 0.71 across the eight scales, which is in the middle of typical ranges of 0.64 to 0.83 (Mehling et al., 2018).

### 4.1. Descriptive results: art appraisal and emotional experience

For an overview of the descriptive statistics of the dataset, see Table 2. We found a wide range of responses for most of the appraisal scales, with means generally above the midpoint, indicating that participants found the artwork relatively interesting (*M* = 4.37) and meaningful (*M* = 4.61) and particularly liked the artwork (*M* = 6.03). The reported experiences of Awe and Perspective change were also positive. For *Transformation* (*M* = 3.29), *Self-reflection* (*M* = 3.87), and especially *Wonder*, did we find notably lower average ratings (*M* = 1.77) with suggestions of a floor effect. Participants generally enjoyed their overall bodily experience (*M* = 5.51).

**Table 2:** Descriptive statistics of artwork ratings.

	**Variable**	**Mean**	**SD**	**Pctl. 25**	**Pctl. 75**
**Appraisals**	Overall enjoyment of bodily experience	5.51	1.55	4.97	6.99
	Perspective change	5.12	1.62	4.54	6.31
	Self-reflection	3.87	1.82	1.22	5.31
	Transformation	3.29	1.76	1.75	4.83
	Awe	4.52	1.85	3.01	6.01
	Wonder	1.77	1.43	1.00	1.83
	Interest	4.37	1.98	2.71	5.91
	Liking	6.03	1.19	6.00	7.00
	Meaning	4.61	1.48	4.00	6.00

### 4.2. Bodily experience

**Figure 3:**
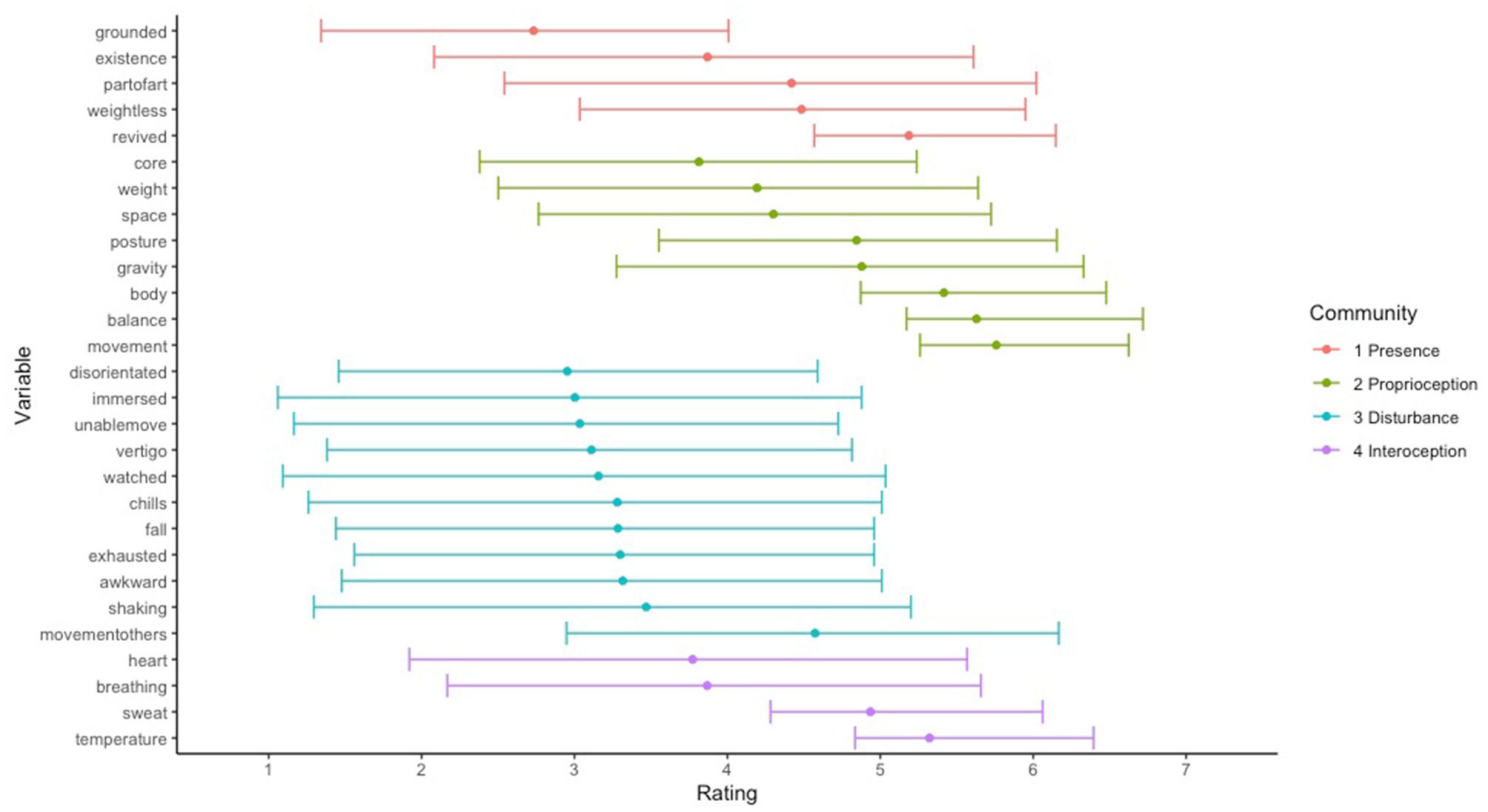
Graph showing means and 95% confidence intervals for the 28 body-items. Note, communities are based on later network analysis.

Descriptive statistics of the means and CI intervals of the different scale-based answers to the body-items are shown in Figure 3. Note that we excluded the item “absorbed” because, during data collection, many German-speaking participants indicated that they did not understand what the term meant in exit interviews, resulting in 28 items. As can be seen, the mean for most scales tended to fall around the midpoint of most scales, with a rather high variance across the participants, and, notably, little indication of major skew or ceiling/floor effects.

The highest noted terms were awareness of movement, balance, participants' overall body, and temperature. The least noted items were feeling grounded, disoriented, and immersed, indicating that participants reported mostly experiences directly related to the awareness of their own body rather than how their body specifically felt or how it felt in relation to the net/other visitors.

#### 4.2.1. Scalar data reduction, network analysis

To clarify the underlying psychological dimensions of bodily responses and their relationships to the experience of the installation art piece, we then used techniques from network science. The application of network science in empirical aesthetics has become more popular in recent years as a tool to discover underlying latent structures in multivariate data (e.g., Pelowski et al., 2018; Coburn et al., 2020; Hayn-Leichsenring et al., 2020; Specker et al., 2021; Weinberger et al., 2021; Christensen et al., 2022). This approach consisted of network construction, followed by community identification and stability analyses. The following analyses were conducted using the *R* software (R Core Team, 2022), and the script is available at the Open Science Framework (OSF) https://osf.io/tgebw/.

##### 4.2.1.1. Network construction

To prepare the body-item dataset, five participants were removed as multivariate outliers via Mahalanobis Distances larger than the critical chi-square value for *df* s at alpha = 0.001 (Mahalanobis, 1936), resulting in a sample of *N* = 230 (140 female, 87 male, one other, two preferred not to answer, *M*_*age*_ = 33.96, *SD* = 14.40, range = 18–72 years) with *N* = 28 body-items. We used the *Triangulated Maximally Filtered Graph* (TMFG; see Massara et al., 2015) to construct the network using the *NetworkToolbox* package (Christensen, 2018) in *R* (see Christensen et al., 2019, 2020; Pelowski et al., 2021 for similar applications). The TMFG does not assume that data are multivariate normal (Golino et al., 2020). Details on the algorithm are in the Supplementary material, and the final TMFG network is in Supplementary Figure 1.

#### 4.2.2. Community identification

We used *Exploratory Graph Analysis* (EGA; Golino and Demetriou, 2017; Golino and Epskamp, 2017) to determine the number of dimensions in the data (i.e., identification of communities of related items) (Golino and Epskamp, 2017), using the used *R* packages *EGAnet* (version 1.1.0; Golino et al., 2022) and *qgraph* (Epskamp et al., 2012). Like principal component or factor analysis, this approach offers data reduction abilities while, in addition providing information about centrality, interconnection, and relative importance of items, allowing for the visualization of the entire network. EGA uses network psychometrics and has been shown to be less affected by aspects such as unidimensionality, interfactor correlations, and sample size, as well as to be more accurate than traditional methods of dimension reduction and factor analysis (e.g., Scee test, parallel analysis, K1 rule; Golino and Demetriou, 2017; Golino and Epskamp, 2017; Golino et al., 2020). This is followed by “community” or dimension identification in the network. For this, we applied the walktrap algorithm via the *igraph* package (Csárdi and Nepusz, 2006) in *R*, which uses “random walks” or a certain number of random “steps” or from one node to another. The algorithm is deterministic and data-driven (i.e., communities are discovered without the researcher's guidance (Christensen and Golino, 2021). Analyses on structural consistency are reported in Supplementary material.

#### 4.2.3. Network model results

**Figure 4:**
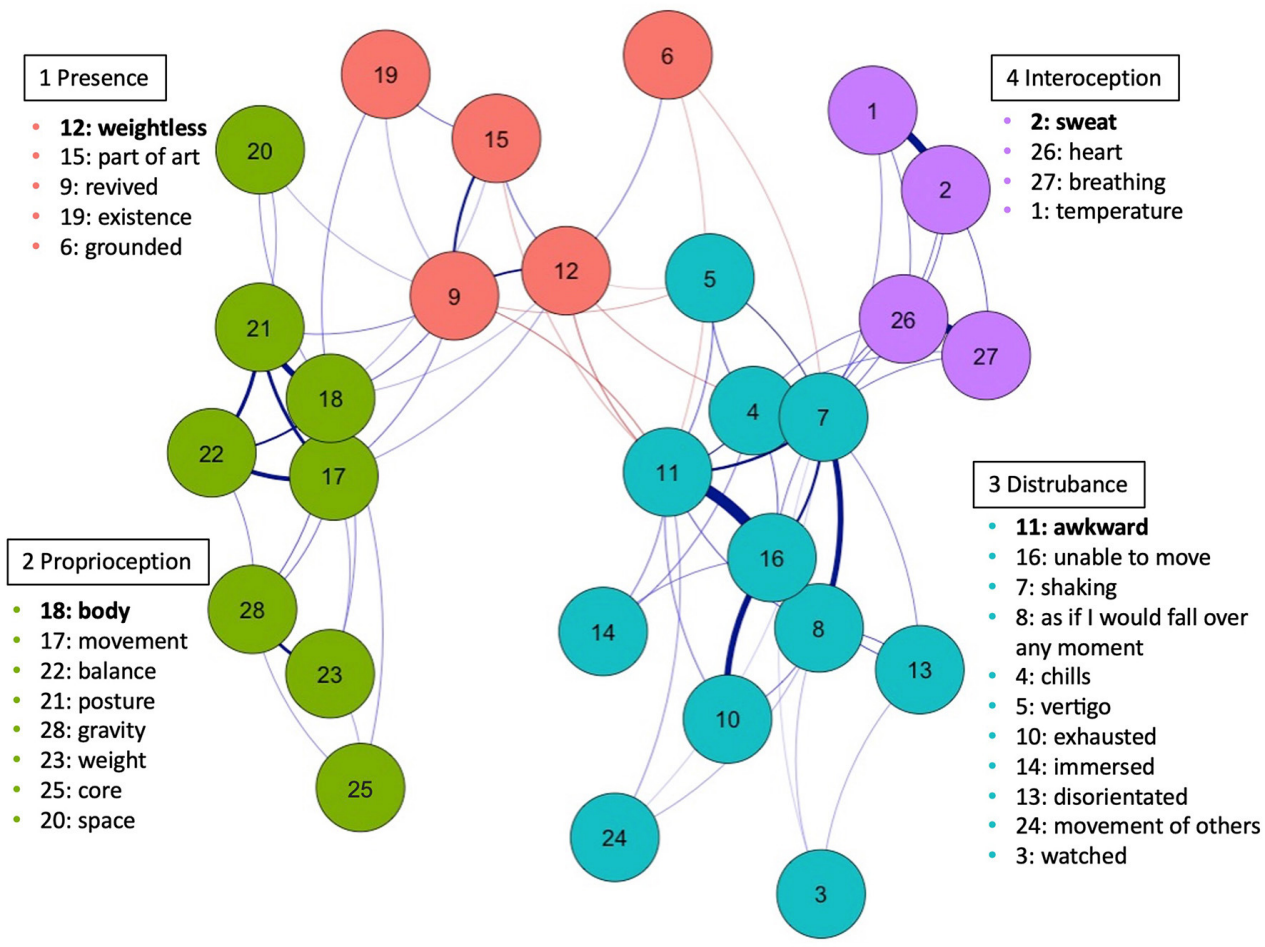
Exploratory graph analysis (EGA) dimensional structure of the body items.

The final network is shown in Figure 4. Nodes, indicated by the colored and numbered circles, represent the measurement items. Nodes are connected by unidirectional edges which represent associations between items (i.e., zero-order correlation surviving the TMFG algorithm). Blue lines indicate positive relations, red lines indicate negative, and thickness indicates the strength of relations.

Network loadings, computed via the *net.loads* function from the EGAnet package, which computes the between- and within-community strength of each item for each community, are shown in Table 3 (highest loadings in bold). The network identified four communities (discussed in descending order, starting with the highest loading items printed in bold per community): (1) a community of four items that generally represented **“Presence”** (i.e., felt presence either within one's own body or feeling a more out-of-body, transcendent state), including feeling “*weightless*,” “grounded,” “revived,” and “part of the art” (mean of the items of Community 1 = 4.14, *SD* = 1.99); (2) eight items that represented **“Proprioceptive experiences”**—awareness of the “*body*,” “movement,” “existence,” “space,” “posture,” “balance,” “weight,” “core,” and “gravity” (*M* = 4.85, *SD* = 1.77); (3) 11 items that described **“Disturbed experiences”**—feeling “*awkward*,” “watched,” “chills,” “vertigo,” “exhausted,” “disorientated,” “shaking,” “immersed,” “unable to move,” the “movement of others,” and being about to “fall” (*M* = 3.32, *SD* = 1.95); and (4) 4 items represented **“Interoceptive experiences”**—feeling one's “*sweat*,” “temperature,” “heart beating,” and “breathing” (*M* = 4.47, *SD* = 1.9). Note that, as with principal component analysis, the naming of components requires some degree of interpretation from the researchers, and thus the bolded labels represent our own best attempt to provide a general label to the collection of items.

**Table 3:** Exploratory graph analysis (EGA) network loadings per community.

**Item**	**1 Presence**	**2 Proprioception**	**3 Disturbance**	**4 Interoception**
**Sweat**			0.07	**0.50**
Heart			0.20	**0.49**
Breathing			0.12	**0.38**
Temperature			0.06	**0.34**
**Awkward**	−0.37		**0.67**	
Unable to move			**0.66**	0.23
Shaking	−0.14		**0.59**	0.49
Fall			**0.51**	
Chills			**0.43**	0.21
Vertigo	−0.10		**0.26**	
Exhausted			**0.26**	
Immersed			**0.24**	
Disorientated			**0.18**	
Movement of others			**0.11**	
Watched			**0.09**	
**Weightless**	**0.41**	0.10	−0.04	
Part of art	**0.40**	0.04	−0.15	
Revived	**0.39**	0.29	−0.10	
Existence	**0.24**	0.08		
Grounded	**0.09**		−0.07	
**Body**	0.34	**0.73**		
Movement	0.19	**0.56**		
Balance		**0.46**		
Posture	0.10	**0.45**		
Gravity		**0.45**		
Weight		**0.32**		
Core		**0.19**		
Space	0.06	**0.13**		

Bold text of the items marks the highest loading. Bold text of the loadings indicates the Community in which the item was placed by the exploratory graph analysis (EGA).

### 4.3. Relationship between body experience communities and art experience

For subsequent analysis, we then computed network scores per participant to represent each individual's placement in each community, based on each node's strength within each Community (i.e., factor) in the network using the *net.scores* function from the EGAnet package. These values are used as network “factor loadings” for the weights of each item (Christensen and Golino, 2021). Notably, network analysis allows nodes to contribute to more than one community, i.e., the network scores account for cross-loadings in their estimation of scores. These loadings are considered in the network scores.

To assess the role that the communities of bodily experiences had on art experience, we then used the participants' network scores in multiple regressions to predict specific art experiences (*Awe, Wonder, Interest, Liking, Meaning, Self-reflection, Perspective Change*, and *Transformation*) (see Table 4 for following results). There was no substantive multi-collinearity in any of the models (all VIFs were < 3.70).

**Table 4:** Summary of regression analysis for art experience.

**Variable**	**Community**	***B***	**SE_β_**	**β**	***t***	**Sig. (*p*)**
Awe	(Intercept)	4.52	0.11	0.00	39.39	**< 0.001** ^**^
	1 Presence	0.18	0.17	0.10	1.05	0.294
	2 Proprioception	0.34	0.21	0.18	1.65	0.100
	3 Disturbance	0.65	0.22	0.35	3.00	**0.003** ^**^
	4 Interoception	0.05	0.18	0.03	0.28	0.778
Liking	(Intercept)	6.03	0.07	−0.00	81.50	**< 0.001** ^**^
	1 Presence	−0.02	0.11	−0.02	−0.18	0.857
	2 Proprioception	0.46	0.13	0.38	3.47	**0.001** ^**^
	3 Disturbance	0.01	0.14	0.01	0.10	0.923
	4 Interoception	−0.04	0.12	−0.04	−0.36	0.715
Meaningful	(Intercept)	4.61	0.09	0.00	50.03	**< 0.001** ^**^
	1 Presence	−0.02	0.14	−0.02	−0.17	0.863
	2 Proprioception	0.52	0.17	0.35	3.13	**0.002** ^**^
	3 Disturbance	−0.11	0.17	−0.07	−0.61	0.542
	4 Interoception	0.12	0.15	0.08	0.84	0.404
Interest	(Intercept)	4.37	0.12	0.00	36.41	**< 0.001** ^**^
	1 Presence	−0.18	0.18	−0.09	−0.98	0.327
	2 Proprioception	0.87	0.21	0.44	4.05	**< 0.001** ^**^
	3 Disturbance	0.08	0.23	0.04	0.35	0.726
	4 Interoception	0.45	0.19	0.23	2.39	**0.018** ^**^
Self-reflection	(Intercept)	3.87	0.11	−0.00	34.27	**< 0.001** ^**^
	1 Presence	0.04	0.17	0.02	0.21	0.836
	2 Proprioception	0.57	0.20	0.32	2.84	**0.005** ^**^
	3 Disturbance	0.45	0.21	0.25	2.12	**0.035** ^**^
	4 Interoception	0.18	0.18	0.19	0.01	0.314
Perspective	(Intercept)	5.12	0.10	0.00	51.73	**< 0.001** ^**^
Change	1 Presence	0.17	0.15	0.10	1.11	0.267
	2 Proprioception	0.62	0.18	0.38	3.47	**0.001** ^**^
	3 Disturbance	0.34	0.19	0.21	1.83	0.069
	4 Interoception	−0.18	0.16	−0.11	−1.12	0.263
Transformation	(Intercept)	3.30	0.10	−0.00	51.73	**< 0.001** ^**^
	1 Presence	−0.22	0.16	−0.12	31.69	0.164
	2 Proprioception	1.05	0.19	0.60	−1.40	**< 0.001** ^**^
	3 Disturbance	0.68	0.20	0.39	5.64	**0.001** ^**^
	4 Interoception	0.04	0.39	0.02	3.47	0.800

*B*, unstandardized regression coefficient; *SE*_β_, standard error of the coefficient; β, standardized coefficient. Asterisks indicate significant *p-*values, uncorrected. Bold values indicate significant *p*-values after Bonferroni correction *p* = 0.006 (0.05/8).

Regarding the emotional experiences among them, the combined model significantly predicted *Awe* [*F*_(4, 225)_ = 8.95, *p* < 0.001, *R*^2^ = 0.13, *R*${}_{adjusted}^{2}$ = 0.12]. Specifically, high scorings on Community 3 (*Disturbance*) significantly predicted higher *Awe* (*t* = 3.00, *p* = 0.002). The ratings on *Wonder* had a strong floor effect, with more than half of the participant sample (*N* = 116) not experiencing any wonder, i.e., rating “1” on the Likert scale. Only 11.3% of the sample gave *Wonder* experience ratings above the midpoint of four (*N* = 26). Given this extreme pattern of ratings and a small sample of participant rating it above the midpoint, we did not compute the regression with the *Wonder* ratings.

The art appraisals, *Liking* [*F*_(4, 224)_ = 10.66, *p* < 0.001, *R*^2^ = 0.14, *R*${}_{adjusted}^{2}$ = 0.12], *Meaning* [*F*_(4, 223)_ = 8.69, *p* < 0.001, *R*^2^ = 0.14, *R*${}_{adjusted}^{2}$ = 0.12], and *Interest* [*F*_(4, 225)_ = 11.31, *p* < 0.001, *R*^2^ = 0.17, *R*${}_{adjusted}^{2}$ = 0.15], were significantly predicted by the combined model. *Liking* (*t* = 3.47, *p* = 0.001) and *Meaning* (*t* = 3.13, *p* = 0.003) were significantly predicted by higher scorings on Community 2 (*Proprioception*). Interest was also significantly predicted by high scorings on Community 2 (*Proprioception*) (*t* = 4.05, *p* < 0.001), but also Community 4 (*Interoception*) (*t* = 2.39, *p* = 0.018).

Finally, we ran two multiple regressions looking at the self-reflective/transformative experience factors. We found a collective significant effect between the four communities and *Change in Perspective* [*F*_(4, 225)_ = 10.6, *p* < 0.001, *R*^2^ = 0.16, *R*${}_{adjusted}^{2}$ = 0.14]. Higher scorings on Community 2 (*Proprioception*) significantly predicted higher *Perspective* changing experiences (*t* = 4.27, *p* < 0.001). *Self-reflection* [*F*_(4, 225)_ = 8.48, *p* < 0.001, *R*^2^ = 0.13, *R*${}_{adjusted}^{2}$ = 0.12] was predicted by Community 2 (*Proprioception*) (*t* = .83, *p* = 0.005) and Community 3 (*Disturbance*) (*t* = 2.12, *p* = 0.035). In the same way, *Transformation* [*F*(4,225) = 15.11, *p* = 0.001, *R*^2^ = 0.21, *R*${}_{adjusted}^{2}$ = 0.20] was predicted by Community 2 (*Proprioception*) (*t* = 5.64, *p* < 0.001) and Community 3 (*Disturbance*) (*t* = 3.47, *p* = 0.001).

### 4.4. Enjoyment of bodily experience and relation to communities and art experience

The combined model assessing the impact of the network dimensions on the enjoyment of the overall bodily experience was also significant [*F*_(4, 225)_ = 0.71, *p* < 0.001, *R*^2^ = 0.42, *R*${}_{adjusted}^{2}$ = 0.41] (see Supplementary Table 3 for full results). Specifically, enjoyment of the bodily experience was predicted by Community 2 (*Proprioception*; *t* = 5.53, *p* < 0.001), Community 3 (*Disturbance*; *t* = −3.01, *p* = 0.003), and Community 4 (*Interoception*; *t* = 2.02, *p* = 0.045). These results indicated that participants enjoyed interoceptive and proprioceptive experiences, but not the experiences that were grouped as disturbing.

To explore the relationship between the enjoyment of the overall bodily experience and art experience, we ran correlations between them [with a Bonferroni adjusted alpha level of 0.008 per test (0.05/6)]. We found a significant positive correlation between overall bodily enjoyment and *Liking*, r_P_(221) = 0.41, *p* < 0.001, 95% CI of correlation (0.29, 0.51), *Meaning*, r_P_(220) = 0.37, *p* < 0.001, 95% CI of correlation (0.25, 0.47), *Interest*, r_P_(222) = 0.24, *p* = 0.001, 95% CI of correlation (0.12, 0.36), *Self-Reflection*, r_P_(221) = 0.16, *p* = *0*.022, 95% CI of correlation (0.03, 0.28), *Perspective change*, r_P_(221) = 0.29, *p* < 0.001, 95% CI of correlation (0.17, 0.40), and *Transformation*, r_P_(233) = 0.23, *p* < 0.001, 95% CI of correlation (0.11, 0.35), indicating that participants who enjoyed the bodily experience also liked the art more, found it more meaningful and interesting, reported that it made them reflect about themselves and their perspectives and that their experience was transformative.

### 4.5. The role of trait interoceptive bodily awareness in art experience

#### 4.5.1. Relationship between trait interoceptive bodily awareness and body experience communities

We then focused on the relation between trait interoceptive bodily awareness (the total score of the MAIA-2 questionnaire) and the reported bodily experience. Simple linear regression analysis indicated that participants who were higher in interoceptive bodily awareness (total MAIA-2 score) scored lower on Community 3 (*Disturbance*) [*F*_(1, 225)_ = 5.04, *p* = 0.026, *R*^2^ = 0.02, *R*${}_{adjusted}^{2}$ = 0.12]. Interoceptive bodily awareness did not affect the other three communities.

For exploratory purposes, to follow-up on the effect of the total interoceptive bodily awareness score (MAIA-2) on scorings in Community 3 (*Disturbance*), we further examined the relationship between the eight subscales of the MAIA-2 and Community 3 (*Disturbance*). The MAIA-2 subscales allow capturing finer dimensions of interoception (see Methods for the description of each subscale). The adjusted alpha level following the Bonferroni correction was set to 0.006 (0.05/8). Community 3 (*Disturbance*) positively correlated “Body Trusting” subscale, *r*_P_(228) = −0.31, *p* = 0.001, 95% CI of correlation (−0.32, −0.08), which indicates seeing one's body as a safe place and trusting one's sensations. All other subscales showed a non-significant correlation to Community 3 (*Disturbance*).^10^ This result indicated that participants who tended to experience their body as safe and trustworthy to a lesser degree have experiences associated with disturbing experiences [i.e., high scorings on Community 3 (*Disturbance*)].

#### 4.5.2. Role of trait interoceptive bodily awareness in the relationship between art experience and communities

Next, we assessed whether relatively more awareness of one's body (MAIA-2) might modulate how we attend to bodily experience and in turn, magnify art appreciation (Jung et al., 2017; Brinck, 2018; Schino et al., 2021). To address this question, we investigated whether MAIA-2 moderated the relationship between the four Communities and art appraisal (*Liking, Interest*, and *Meaning*) by adding body awareness as an interaction term with the Communities to the regression models predicting appraisal. Interoception did not moderate the relationship between the four Communities and the appraisal ratings (see Supplementary Table 4 for full results).

### 4.6. What do visitors spontaneously report about bodily experiences with installation art?

As a last step, we looked at the open-ended answers to further address our first research question regarding what bodily experiences visitors report.

#### 4.6.1. Qualitative content analysis

A total of 235 qualitative records from the final participant sample were obtained (see descriptive demographic statistics in Supplementary Table 1), with 36 records not used for the analysis because responses did not mention experiences related to the body, which was specially asked for in the open question, or participants responded with only a single word, leaving a sample of *N* = 199 for the qualitative analysis. Each response included, on average two mentions of experiences related to the body. Response data were analyzed in a bottom-up manner using inductive content analysis (ICA), a form of qualitative content analysis (Thomas, 2006; Bengtsson, 2016; Kyngäs, 2020; Bingham and Witkowsky, 2022).

Table 5 shows the final 12 categories of bodily experiences reported by the visitors. The semantic categories are depicted in decreasing order of occurrence in the descriptions collected.

**Table 5:** Coding scheme to categorize visitors' bodily experience of the installation, characteristic terms, and frequency.

**Category**	**Community**	***N***	**%**	**Criteria**	**Sample responses**
Awareness of movement	2	51	25.63%	Reflecting about steps/movement/body coordination/body control/type of movement (e.g., bent over, upright, slow, like a spider), awareness of movement type: laying, sitting, crawling, climbing, small steps, upright/bent position	“*I liked the movement on all fours.”; “[...] intuitive motions, like sitting down and walking became more reflected.” “Upright locomotion was difficult in part because the net gives way easily. I automatically wanted to hold on and move bent over.”; “Finding balance was the hardest part of the installation. The coordination of one's own body and the movements with what one sees was difficult for me and became apparent in my exhaustion.”*
Temperature	4	46	23.12%	Heat, sweating, warmth, thirst, blood rushing to head	“*I noticed that my body had to work really hard, because after I was out of the installation again, I was hot.”; “Sweaty hands and feet.”*
Body shaking	3	44	22.11%	Body parts shaking, tingling, trembling, shaky, soft knees, legs such as pudding, wobbly knees, unsteady, nervous, restlessness	“*My legs were like pudding and I was shaking slightly.”; “Short uncertainty because of the slopes and the wobbly ground.”*
Body trusting	__	39	19.60%	Trusting one's own body; reflecting about one's own body's capabilities/lack of control; accepting or overcoming one's body's limitations, becoming more confident/self-assured in walking	“*My body brought me into the installation. It accompanied me there and made it possible for me to take one step after the other on the net. When I felt I needed more support, I used my arms and hands to hold on.”; “Towards the end, I became more confident, and I trusted my body more.”*
Physical effort	3	38	19.10%	Physical effort/exhaustion, challenge, overcoming boundaries, uneasiness	“*[...] overcoming one's own barriers”; “Physical border experience expressed in rapid breathing and soft legs.”;*
Awareness of body weight	1/2	38	19.10%	Weightlessness, lightness, flying, hovering, floating, being in space, being carried/captured, feeling heavy/gravity/one's weight, flying	“*I was often thinking of my own physical weight and position on the wire netting. Even more so when directly over the highest section of the atrium.” “I felt free, and with a different perception of gravity.”*
Balance/body tension	2	29	14.57%	Being aware of/keeping balance, sense of equilibrium, stability, muscle tension	“*Due to the soft and different yielding ground, many different muscle groups are used for stabilization.”; “It was like floating in the air...together with the feeling of having to be super mindful of every muscle in the body. Almost meditative.”*
Awareness of body in relation to net	__	24	12.06%	Feeling the net adapting to one's body weight, awareness of the body in relation to net, noticing how the body moved differently due to givens of net, focus on the material of installation	“*One is aware of one's size and shape in relation to the net. How one sets oneself apart, intervenes, fits in.”; “My body felt securely supported by the net. The soft and elastic surface allowed me to assess my body weight much better than on solid ground and I realized that I am heavier than I usually thought.”; “I focused on the metal netting...”*
Revival/adrenaline	1	21	10.55%	Awake, revival, reactive, euphoria, energy, elation, excitement, arousal, alert, adrenaline	“*Adrenaline”; “euphoria”*
Vertigo	3	19	9.55%	Vertigo; feeling dizzy, malaise, nausea, fluttering/fear/tension/lump/funny feeling in the stomach	“*Vertigo at first, then lightness of the body.”; “Dizziness and strong tingling in the abdomen when looking down.”*
Heartbeat	4	18	9.05%	Heartbeat, palpitations, rapid pulse	“*My heart was pounding wildly.”; “[...] heartbeat was faster,”*
Resistance	3	13	6.53%	Feeling unable to move, tense, stiff, paralyzed; body resists	“*Although I could move freely, my body felt shaky and reluctant.”; “Not wanting to move away from a stable point.”*
Awareness of other visitors	3	12	6.03%	Feeling other people's movements; feeling the net move, feeling vibrations through the others	“*Noticing and feeling the movement of the other participants was interesting and influenced me in my approach from time to time.”; “You learn very quickly how to move and you notice from the vibrations when other people enter the net.”*
New/heightened body experience	__	11	5.53%	General body awareness intensified/other body feelings	“*I felt aware of my body in a way I didn't before.”; “intensified body sensation”*
Breathing	4	8	4.02%	Being aware of breathing/noticing faster breath	“*[...] breathing got faster”; “[…] fast breathing [...]”*

Community numbers correspond to 1 = *Presence*; 2 = *Proprioception*; 3 = *Disturbance*; 4 = *Interoception*. The category “Awareness of Body Weight” was categorized both into Communities 1 (*Presence*) and 2 (*Proprioception*) because it addresses awareness of weight (Community 2 *Proprioception*), as well as feeling weightless (Community 1 *Presence*).

##### 4.6.1.1. Example written responses for communities

To connect the results of our qualitative analysis with our quantitative analysis, we categorized the qualitative categories into the four Communities from the network analysis where applicable (see row “Community” in Table 5) after we had analyzed the quantitative data. For these communities, written examples of what the experience type based on the community might have felt like for the participants are given in the last column. Three categories (body trusting, new/heightened body experience, and awareness of the body in relation to the net) were not explicitly captured by our communities. Thus, we specifically focus on these categories in the following.

Many participants reflected on their trust in and abilities of their body (*N* = 39, labeled “Body Trusting”), with many reporting that they were able to overcome physical limitations or challenges over time and by trusting their own bodies. Based on the exploration of the MAIA-2 subscales in our previous analysis, we assume that participants who tended to experience their bodies as safe and trustworthy tend to have a more positive and less disturbing/distracting art experience.

Eleven participants reported that they had new or intensified bodily experiences, e.g., “*I felt aware of my body in a way I didn't before”*. While we do assume that the body trusting category was rather largely based on the nature of the case installation artwork, we would suggest including an assessment on whether visitors experience new or heightened awareness/feelings of their body in future assessments of installation art, which he had not been captured in our items but seems to be an important aspect, which has been described with other installation artworks. For example, Kessels and Schrenk (2022) discuss the installation *Tight Roaring Circle* (1997) by Dana Caspersens, William Forsythes, and Joel Ryans, which consists of a giant white bouncy castle at the Roundhouse London. Once entering the bouncy castle, the most natural movements become mannered and thus palpable: the installation gives visitors the opportunity to experience *new* proprioceptive and interoceptive sensations.

Interestingly, a fair number of participants (*N* = 24) reflected upon their physical relation to the net, such as how it responds to one's moving in terms of giving in to weight and shaking or how one's body size fits in (e.g., “*One is aware of one's size and shape in relation to the net. How one sets oneself apart, intervenes, fits in”)*. One individual specifically reported that they enjoyed feeling the vibrations while lying on the net (“*I like that I can feel the vibrations of others while lying down*”). This experience type would be mostly related to feeling a “part of the art,” which was part of Community 1 (*Presence*). However, besides the participatory aspect, we want to emphasize that this category summarizes an important aspect of the experience of installation art that we did not capture in our review above yet, which is that visitors engage with and are aware of the physical properties of the artwork in relation to their own bodies. This illustrates that installation art is not viewed from a distance but is always experienced in relation to one's body and interaction with it, which should be included in future assessments of installation art.

## 5. Discussion

This study outlined the ways the body is addressed in installation art and discussed its role in the appreciation of such artworks. Overall, we identified a significant need to consider and empirically assess the role of the body in the experience of installation art. We aimed to fill that gap by capturing visitors' subjective bodily experiences in an installation art piece (Tomás Saraceno's installation *in orbit*) using a mixed-methods design (qualitative content analysis and network analysis of quantitative data) to explore the relationship between bodily experience, individual differences in body awareness, and art experience.

We chose 28 body experience items to capture which kinds of subjective bodily experiences individuals might report and could group them into four communities using a network modeling approach to illustrate their connections. Two communities entailed body-items related more to bodily positions and movements of the body vs. inner aspects. Experiences related to sensing one's outer body and movements were categorized as *Proprioception* (Community 2) (i.e., perception of muscle tensions, movement, posture, and balance), while experiences related to sensing one's inner body were termed *Interoception* (Community 4) (i.e., perception of sensations from inside the body related to internal organ function, such as heartbeat and respiration; Mehling et al., 2009). Community 3 (*Disturbance*) involved items that might especially cause a disturbance or rupture of one's bodily schema in terms of, e.g., stability and orientation, potentially allocating an attentional switch to the conscious experience of one's body while being in the installation (Jelić et al., 2016). Finally, Community 1 (*Presence*) reflected experiences capturing the important element of participation and how installation art can make one aware of one's existence and presence in the space (Noë, 2000; Bishop, 2005).

Regarding the role of the communities in the art experience, we found that especially *Proprioception* (Community 2) played an important role in the evaluation of the art (*Interest, Liking*, and *Meaning*) and transformative outcomes (*Self-reflection, Perspective Change*, and *Transformation*). Thus, we recommend that future research in installation art should assess experiences related to Community 2 *Proprioception* as it shows relations to art appreciation. Using our suggestion, future studies ought to test the hypothesis that more reported proprioceptive experiences, above other experiences, lead to more intense and impactful art experience, as suggested in our theoretical and anecdotal review (Best, 2002; Bishop, 2005; Montero, 2006; Lanzoni, 2009; Rebentisch, 2012; Shusterman, 2012).

Interestingly, Community 3 *Disturbance* predicted *Transformation*. This finding supports theoretical arguments that suggest a key role of disruption within the process of transformation in art experience (Pelowski et al., 2017; Kühnapfel and Fingerhut, forthcoming). In this line, we also found that Community 3 *Disturbance* predicted *Awe*, an emotion that had been specifically associated with self-relevance and transformation (Keltner and Haidt, 2003; Shiota et al., 2007; Schneider, 2009; Cohen et al., 2010; Silvia and Nusbaum, 2011; Yaden et al., 2017; Fingerhut and Prinz, 2018, 2020). Transformation may especially occur in situations where there is a new relation to the environment and routines become disturbed and not fluent. Indeed, the kind of movement the installation affords in the net can be in such a way function as a bodily disturbance. Furthermore, certain “triggers”, such as feeling chills or being watched, can shift the visitors' attention to the self, inducing reflection (Pelowski, 2015). In addition, a kind of physical intervention or even rupture of our body in the installation (Jelić et al., 2016; Jelić and Staničić, 2021) might be central for a deeper awareness during art engagement and thus for transformative outcomes. It might be that, after all that, the way installation addresses the body “interrupts, challenges and engages us in a way that is directed at something beyond the ordinary” and induces a “perspectival change that we value in art” (Fingerhut, 2018, p. 87).

All four communities based on the network analysis were also captured in the open-ended questions, indicating that they are part of the art experience. Thus, future studies assessing the art experience of installation art should at least assess some items pertaining to all four communities to capture a broad range, as well as more disturbing or negative experiences (see Community 3 *Disturbance*) if interested in transformative outcomes. In addition, we suggest assessing experiences related to new or heightened bodily experience and how one feels in relation to the artwork and space, which were revealed to be essential experience aspects via the complementary bottom-up qualitative but not directly captured in our top-down body items/communities.

Regarding emotional experience, surprisingly, we found a floor effect of wonder ratings. It has been argued that many emotions, including wonder, have specific embodiment or somatic profiles (Fingerhut and Prinz, 2018, 2020). Wonder, as examined in studies on emotional faces (e.g., Feleky, 1914) or depictions of wonder in artworks (e.g., *Self-Portrait* by Franz Xaver Messerschmidt, 1976), may yield a slack jaw, widening of eyes, a slight lift of brows, or a head lift. To feel wonder, one might thus have to remain rather static and transfixed (Fingerhut and Prinz, 2018) to save processing resources for the high perceptual and cognitive engagement that are typical of wonder (also see “stopping for knowledge” and similar arguments (Sarasso et al., 2020; Fingerhut and Kühnapfel, forthcoming). Similarly, it has been argued that high body awareness may also be contrary to absorbing, harmonious, and wonderous experiences (Pelowski and Akiba, 2011). One fascinating outcome of our study was that we found a possible dissociation of awe from wonder that is often treated as similar in the literature. While participants gave overall very low ratings for wonder (*M* = 1.77), the awe ratings were relatively high (*M* = 4.52). As we have argued, wonder might have a more cognitive sub-emotional component than the more straightforward, overwhelming awe (Fingerhut and Prinz, 2018). This form of cognitive complexity or challenge might lack from our installation art piece constitutes a rather bodily challenge. Similar things might be said regarding the spiritual sub-emotional component of wonder: veneration might not relate as well to the playful engagement required to engage Saraceno's work. Future research with installation art needs to refine this relationship between bodily involvements and wonder experiences.

We assessed whether participants who generally attend to their body more than others (as measured by the MAIA-2) report more/specific bodily experiences. We found that the body trusting subscale drove the only found effect of the MAIA-2 on Community 3 (*Disturbance*). This indicates that participants who generally feel less safe in their bodies also rated the *Disturbance* items higher. We note that this effect is very specific to the installation, which makes visitors deal with the fear of height and trusting their body in the net, which suggests that individual differences in the MAIA-2 might not be related to overall bodily art experience other than those related to height and insecurity characteristics of the case installation. This effect also fits our finding that participants who enjoyed their bodily experience reported higher liking and meaning, indicating a relationship between enjoying/appreciating one's bodily experience and art evaluation. Furthermore, this suggests that body awareness/experience that is uncomfortable or not enjoyed might also have a negative outcome, as has been argued previously: if individuals feel rather uncomfortable, unsure, or insecure in a situation, this might be enhanced by body awareness and lead to reduced attention on the artwork (Pelowski et al., 2014).

We also found no moderation of the MAIA-2 score on the relationship between the communities and art experience, also suggesting no role of individual differences in body awareness for art experience. This null result might also be because the MAIA-2 is mainly concerned with inner bodily sensations. Nevertheless, we chose MAIA-2 to be the best fit for assessing body awareness among available body awareness trait questionnaires, which focus on the awareness of internal bodily sensations (Miller et al., 1981), sensitivity to bodily processes (Shields et al., 1989), subjective experiences of organs and the autonomic nervous system (Porges, 1993), or the tendency to integrate body sensations into conscious awareness to guide decision making and behavior (Daubenmier, 2005, also see Three-domain Interoceptive Sensations Questionnaire, Vlemincx et al., 2021; Interoceptive Confusion Questionnaire, Brewer et al., 2016). This also calls for a validated survey assessing general body awareness, also covering proprioceptive, kinesthetic, and exteroceptive dimensions besides interoceptive ones. To summarize, whether the appreciation of the bodily experience and bodily participation in the artwork really is an integral part of the art evaluation and assessment of its quality needs to be addressed in future studies using to-be-developed general trait body awareness surveys.

## 6. Caveats, implications for future research, and conclusion

This study has limitations. We aimed to conduct and analyze a unique case study, and our presented results, therefore, cannot generalize to other artworks. However, we are confident that due to our rather large participant sample, we were able to cover a good range of variety in responses.

Thus, future analyses should include a broader range of installation art. We also hope that future studies will formulate more focused hypotheses, such as, e.g., increased body awareness, and relatively more bodily experience could make the installation art encounter more intense, resonant, or memorable, as was suggested in the theoretical literature (e.g., Best, 2002; Oliveira et al., 2003; Robinson, 2012) and based on our finding of the positive association of art experience outcomes (i.e., liking, meaning, and interest) and bodily experiences related to proprioception. Future studies could also assess whether bodily experience differs based on participants' art interest and knowledge levels, which have been shown to impact aesthetic experience with other art forms (see Specker et al., 2020 for review). A unified and validated body experience survey is needed to compare findings from different installation artworks. A further complication comes with the field of embodied aesthetic emotions (such as wonder and interest): engagement and evaluative elements share embodied resources (Fingerhut and Kühnapfel, forthcoming). This can make it hard to identify which bodily responses are necessitated by the artwork (to experience it at all) and which contribute to an embodied evaluation of the artwork. This problem more directly might pertain to installation art.

While our study was survey-based, future studies could also add recordings of the physiological data (e.g., see suggestions in Schino et al., 2022) and track participants' amount of movement (e.g., Kühnapfel et al., 2023) to capture more objective indicators of physical engagement, followed by subjective reports to get insight how specific engagement might have felt.

Future studies could also employ installation artworks that do not evoke such direct and powerful corporeal effects such as by disturbing visitors' balance, stability, and upright position but instead creating experimental conditions by making participants aware of their body (e.g., via a body scan meditation, also see Dekeyzer, 2022 for a new meditation method based on proprioception to experience artworks starting from the body), or instructing one experimental group to specifically attend to their body while experiencing art. Future studies could also assess participants' experience with body exercises (e.g., body scan meditation, yoga). Finally, we did not assess whether participants read the text about the installation artwork in the museum or on the museum website. Future studies should avoid this limitation.

We hope to have shown the value of adding qualitative assessments to the traditional quantitative assessment approach in empirical aesthetics, as the qualitative data revealed bodily experience dimensions that were not yet captured by our top-down selected body-items. Thus, this might help avoid biases on the researcher's side. Our study is an example application of methodological triangulation (i.e., using different methodologies to approach the same topic; Todd et al., 2004). We recommend this mixed-methods design when investigating types of art experiences with installation art or other art genres that have yet to be systematically studied.

Our study also has broader implications. Unlocking the body's role may be important for understanding the impact and value of art. Especially in times of increasing digital presence, installation art offers an in-person interaction and bodily experience, making it potentially a higher valued and comparably intense art experience. Indeed, contemporary art practice has increasingly turned toward the production of *physical* experiences (von Hantelmann, 2014; Kessels and Schrenk, 2022; Spence, 2022)—a movement that has been described as the “sensorial turn” (Levent et al., 2014, p. xvii), “experiential turn” (von Hantelmann, 2014), or “participation age” (Almenberg, 2010, p. 3). Insights on the role of heightened body awareness in experiencing profound or transformative states can help artists, curators, and museums to create more engaging and immersive experiences for visitors.

That said, with our theoretical review, we have made an argument for the central role of the body in the reception and appreciation of installation art. With our *in situ* ecologically valid case study, we showed that people report a wealth of bodily experiences when being asked about their experience, indicating a special role of proprioceptive and disturbing experiences for appreciation, with the latter specifically playing a role in transformative outcomes. Together, we hope to have planted the seed for a future embodied aesthetics of (installation) art that considers the multifaceted embodied responses we described.

## Data availability statement

The raw data supporting the conclusions of this article will be made available by the authors, without undue reservation.

## Ethics statement

The studies involving human participants were reviewed and approved by University of Vienna Ethics Committee. The patients/participants provided their written informed consent to participate in this study.

## Author contributions

CK conceived the study, collected data, preprocessed and analyzed data, and wrote the manuscript. CK, MP, and JF designed the study. MP and JF edited and provided comments on the finalized manuscript. All authors contributed to the article and approved the submitted version.

## Footnotes

^1^Another line of research on embodiment concerns representational forms of the embodiment in the brain, such as the role of motor responses in terms of embodied simulation in art experience (e.g., Freedberg and Gallese, 2007; Umiltà et al., 2012; Finisguerra et al., 2021; so-called “weak embodiment;” Gallagher, 2018). Even though this constitutes an important addition to the study of embodiment, for this paper, we are interested in aspects of the actual bodily engagement and the conscious experience of one's body during art engagement (i.e., “strong” embodiment; also see Matheson and Kenett, 2020; for review on weak and strong embodiment in creativity).

^2^Rebentisch (2012) on the participatory and performative character of installation art: “…the experience of art, including and especially that of committed [installation] art, undermines simple identifications and places the receiving subject in a self-reflective and performative relation to the object” (p. 271).

^3^German original: “Somit wird die Installation nicht nur visuell und haptisch erlebt, auch die Wahrnehmung der Bewegung des eigenen Körpers im Raum und der Anspannung der Muskulatur in Gliedmaßen und im Rumpf sowie mögliche Effekte auf den Herzschlag, die Atmung und das generelle Stresslevel sind integraler Teil der Rezeption des Kunstwerks” (Kessels and Schrenk, 2022, p. 2).

^4^To quote one visitor's testimony from an online article: “These emotional qualities [or the art experience] are not inherent to the material properties of the work but are only produced through audience participation. Therefore, I read this work as a performance event focusing on the role of the spectator's body” (Putnam, 2014).

^5^It has been described the interactive installation gives visitors “the opportunity to perceive the world from new heights and vantage points” (Chin, 2013).

^6^How perspective change is facilitated becomes clear through this quote: “museum visitors who climb up into the net, high above ground level, confront new dimensions of (self)perception through a personal and defining experience” (Divisare, 2013).

^7^The instructions were as follows: “*First, please think about your* ***experience of your own*** ***body*** *when you were engaging with the art. We will ask you about other aspects of your experience later. For now, just think about* ***experiences related to your*** ***body****. Please describe briefly: [open textbox]”*.

^8^In addition, as part of a wider data collection for a different study, we also collected some emotional/phenomenal factors. Also, participants were asked which of the following they would use to label their experience: transformative, novel, negative or bad, harmonious, absorbing, or neutral. These labels were based on theoretical models of art experience types, as well as recent empirical studies which used this question construction, but will not be further considered in the present paper.

^9^We controlled for the impact of whether participants experienced the installation by themselves vs. others, and fear of height on art experience (see Supplementary material for results).

^10^For exploratory purposes, we report two correlations at the *p*>.05 significant level without Bonferroni correction: The correlation between Community 3 and the “Attention Regulation” subscale was significant at *p* < .05: *r*_*P*_(228) = −.15, *p* = 0.022, 95% CI of correlation [−0.27 −0.02]. This result indicates that individuals with a lower ability to sustain and control attention to bodily sensations tended to report experiences associated with Community 3.
