# Supplementary material for: The role of the body in the experience of installation art: a case study of visitors' bodily, emotional, and transformative experiences in Tomás Saraceno's “in orbit”
Source: Front Psychol. 2023 Jul 17;14:1192689. doi: 10.3389/fpsyg.2023.1192689 (PMC10389276; doi:10.3389/fpsyg.2023.1192689)
Supplement: Supplementary file 1 [file Data_Sheet_1.docx]

Supplementary Material

The Role of the Body in the Experience of Installation Art:

A Case Study of Visitors’ Bodily, Emotional, and Transformative Experience in Tomás Saraceno’s *in orbit*

Kühnapfel, C.^*^, Fingerhut. J., Pelowski, M.

*** Correspondence:** Corinna Kühnapfel corinna.kuehnapfel@univie.ac.at

# Supplementary Figures and Tables

## Supplementary Figures

**
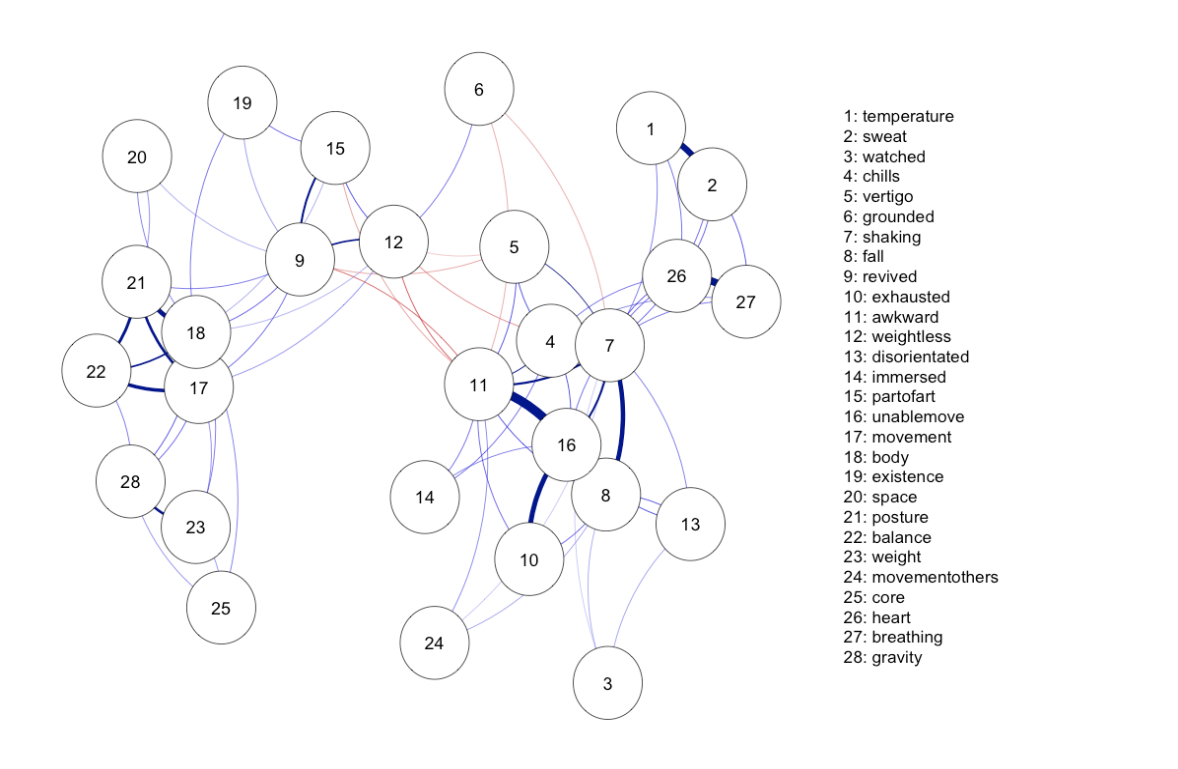
**

**Supplementary Figure 1.** Triangulated Maximally Filtered Graph (TMFG) dimensional structure of the body-items

**
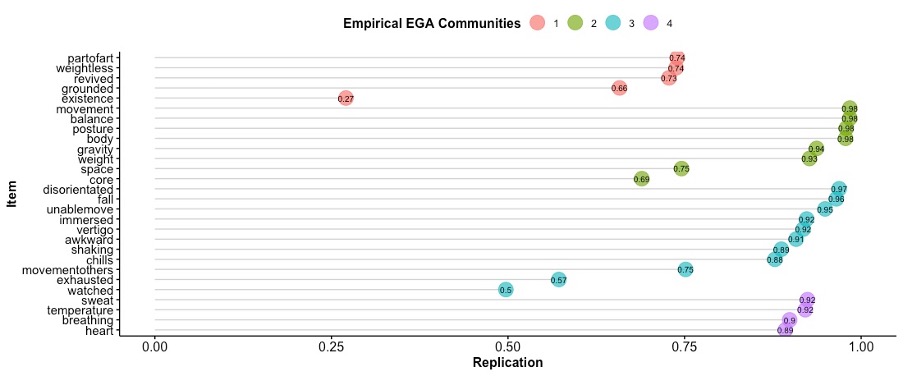
**

**Supplementary Figure 2.** Item stability of body-items (y-axis). Community numbers correspond to 1 = *Presence*; 2 = *Proprioception*; 3 = *Disturbance*; 4 = *Interoception*

**
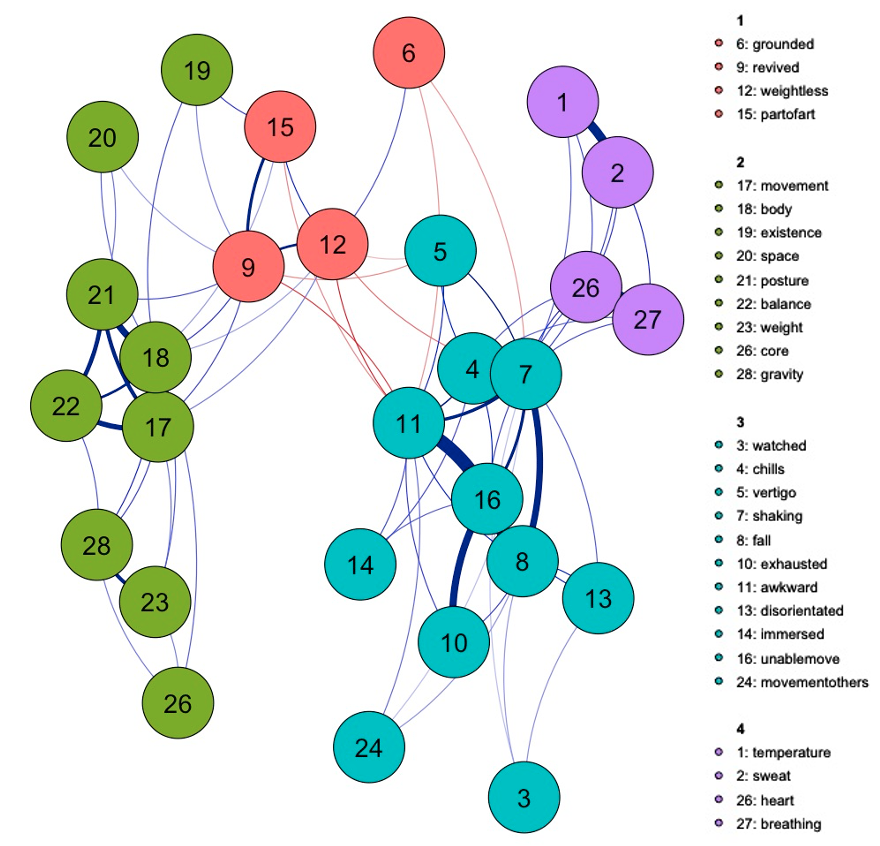
**

**Supplementary Figure 3.** bootEGA dimensional structure of the body-items. Community numbers correspond to 1 = *Presence*; 2 = *Proprioception*; 3 = *Disturbance*; 4 = *Interoception*


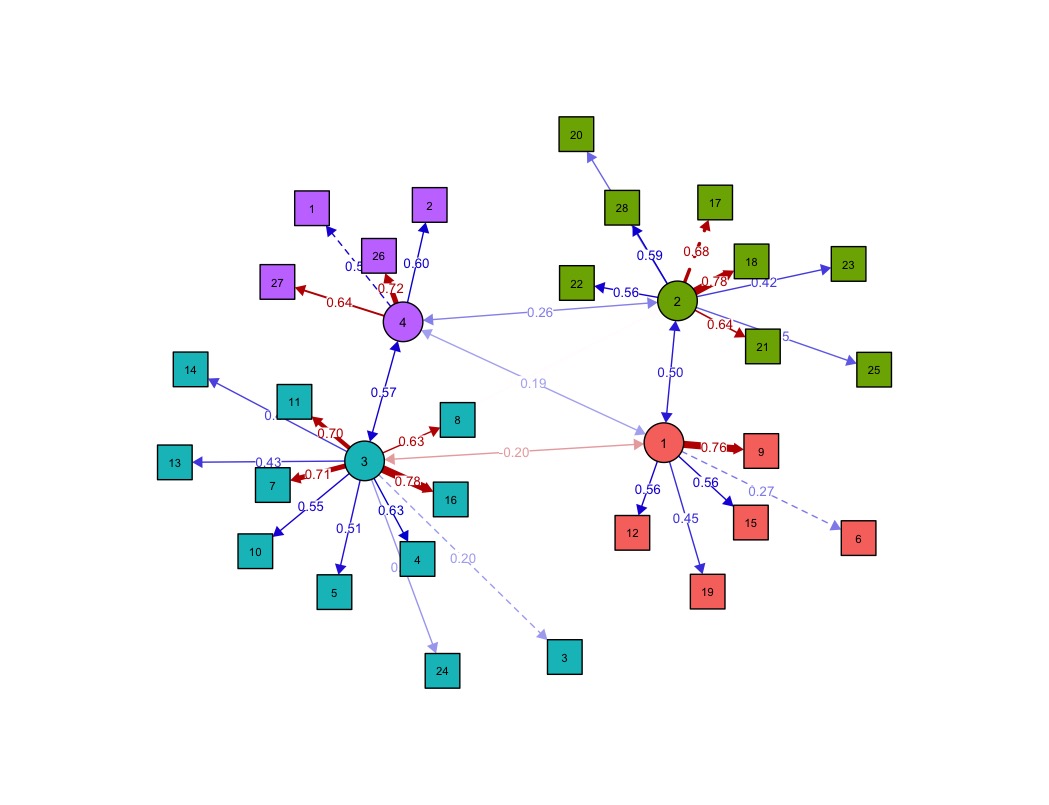


**Supplementary Figure 4.** Confirmatory Factor Analysis (CFA) plot. Community numbers correspond to 1 = *Presence*; 2 = *Proprioception*; 3 = *Disturbance*; 4 = *Interoception*

## Supplementary Tables

**Supplementary Table 1.** Descriptive demographic statistics

|  | **N** | **Mean** | **Std. Dev.** | **Min** | **Pctl. 25** | **Pctl. 75** | **Max** |
| --- | --- | --- | --- | --- | --- | --- | --- |
| Age | 235 | 33.78 | 14.39 | 18 | 22 | 44 | 72 |
| Gender |  |  |  |  |  |  |  |
| ... Female | 142 | 60.4% |  |  |  |  |  |
| ... Male | 89 | 37.9% |  |  |  |  |  |
| ... Other | 2 | 0.9% |  |  |  |  |  |
| ... Prefer not to answer | 2 | 0.9% |  |  |  |  |  |
| Art interest | 234 | 6.23 | 1.163 | 1 | 6 | 7 | 7 |
| No art knowledge | 185 | 78.7% |  |  |  |  |  |
| Art knowledge | 50 | 21.3% |  |  |  |  |  |
| Art engagement/ number of art museum visits |  |  |  |  |  |  |  |
| ... Less than once per year | 31 |  |  |  |  |  |  |
| ... Once every 3 months | 58 |  |  |  |  |  |  |
| ... Once every two weeks | 10 |  |  |  |  |  |  |
| ... Once every week or more often | 4 |  |  |  |  |  |  |
| ... Once in half a year | 69 |  |  |  |  |  |  |
| ... Once per month | 25 |  |  |  |  |  |  |
| ... Once per year | 38 |  |  |  |  |  |  |
| Like installation art | 233 | 6.31 | 1.09 | 1 | 6 | 7 | 7 |
| Fear of heights | 235 | 4.39 | 2.19 | 1 | 3 | 6 | 7 |
| First time experience | 196 | 83.4% |  |  |  |  |  |
| Experienced it before | 39 | 16.6% |  |  |  |  |  |
| … How many times? | 20 | 1.55 | 0.69 | 1 | 1 | 2 | 3 |
| Experienced by myself | 26 | 11.1% |  |  |  |  |  |
| Experienced with friends/family/ colleagues etc. | 196 | 83.4% |  |  |  |  |  |
| Noticing | 4.29 | 0.63 | 4.00 | 4.75 |  |  |  |
| Not-Distracting | 1.28 | 0.87 | 0.60 | 1.80 |  |  |  |
| Not-Worrying | 2.17 | 0.93 | 1.60 | 2.80 |  |  |  |
| Attention Regulation | 3.78 | 0.73 | 3.43 | 4.43 |  |  |  |
| Emotional Awareness | 4.48 | 0.58 | 4.20 | 5.00 |  |  |  |
| Self-Regulation | 3.73 | 0.86 | 3.23 | 4.50 |  |  |  |
| Body Listening | 3.44 | 1.04 | 2.67 | 4.30 |  |  |  |
| Trusting | 4.25 | 0.82 | 4.00 | 5.00 |  |  |  |
| MAIA-2 total score | 3.40 | 4.40 | 3.06 | 3.74 |  |  |  |

**Supplementary Table 2.** Means, standard deviations, and correlations with confidence intervals of art appraisals collected in the study. Only Interest, Meaning, and Liking fed into the analysis of this study.

| Variable | *M* | *SD* | 1 | 2 | 3 | 4 | 5 | 6 | 7 | 8 | 9 | 10 |
| --- | --- | --- | --- | --- | --- | --- | --- | --- | --- | --- | --- | --- |
| **1. Liking** | 6.03 | 1.20 |  |  |  |  |  |  |  |  |  |  |
| **2. Interest** | 5.00 | 1.49 | .61** |  |  |  |  |  |  |  |  |  |
|  |  |  | [.52, .69] |  |  |  |  |  |  |  |  |  |
| **3. Meaning** | 4.61 | 1.48 | .53** | .64** |  |  |  |  |  |  |  |  |
|  |  |  | [.43, .62] | [.56, .71] |  |  |  |  |  |  |  |  |
| 4. Understanding | 5.70 | 1.20 | .69** | .56** | .57** |  |  |  |  |  |  |  |
|  |  |  | [.62, .76] | [.46, .64] | [.48, .65] |  |  |  |  |  |  |  |
| 5. Good | 5.52 | 1.45 | .57** | .58** | .49** | .62** |  |  |  |  |  |  |
|  |  |  | [.47, .65] | [.49, .66] | [.38, .58] | [.53, .69] |  |  |  |  |  |  |
| 6. Beautiful | 5.19 | 1.60 | .32** | .39** | .24** | .25** | .32** |  |  |  |  |  |
|  |  |  | [.20, .43] | [.27, .49] | [.11, .35] | [.13, .37] | [.20, .43] |  |  |  |  |  |
|  |  |  |  |  |  |  |  |  |  |  |  |  |
| 7. Complex | 4.94 | 1.68 | .45** | .48** | .41** | .41** | .44** | .25** |  |  |  |  |
|  |  |  | [.34, .55] | [.37, .57] | [.29, .51] | [.29, .51] | [.33, .54] | [.13, .37] |  |  |  |  |
| 8. “I would pay to see this art again.” | 4.91 | 1.73 | .47** | .53** | .42** | .49** | .46** | .31** | .52** |  |  |  |
|  |  |  | [.36, .57] | [.43, .62] | [.31, .52] | [.38, .58] | [.35, .56] | [.19, .42] | [.42, .61] |  |  |  |
| 9. “The art made me feel better.” | 3.04 | 1.74 | .20** | .42** | .35** | .28** | .32** | .16* | .25** | .49** |  |  |
|  |  |  | [.07, .32] | [.30, .52] | [.23, .46] | [.16, .40] | [.20, .43] | [.03, .29] | [.12, .37] | [.38, .58] |  |  |
| 10. “The art made me a better person.” | 3.06 | 1.64 | .24** | .47** | .35** | .22** | .32** | .14* | .29** | .43** | .70** |  |
|  |  |  | [.11, .36] | [.37, .57] | [.23, .46] | [.09, .34] | [.19, .43] | [.01, .26] | [.16, .40] | [.32, .53] | [.63, .76] |  |
| 11. “The art changed me.” | 6.03 | 1.20 | 1.00** | .61** | .53** | .69** | .57** | .32** | .45** | .47** | .20** | .24** |
|  |  |  | [1.00, 1.00] | [.52, .69] | [.43, .62] | [.62, .76] | [.47, .65] | [.20, .43] | [.34, .55] | [.36, .57] | [.07, .32] | [.11, .36] |

*Note.* *M* and *SD* are used to represent mean and standard deviation, respectively. Values in square brackets indicate the 95% confidence interval for each correlation. The confidence interval is a plausible range of population correlations that could have caused the sample correlation (Cumming, 2014). * indicates *p* < .05. ** indicates *p* < .01, uncorrected.

**Supplementary Table 3.** Summary of regression analysis for overall bodily enjoyment

| Variable | Community | *B* | *SE_β_* | *β* | *t* | ***Sig. (p)*** | |
| --- | --- | --- | --- | --- | --- | --- | --- |
| enjoyment of overall bodily experience | (Intercept) | 5.51 | 0.08 | -0.00 | 70.05 | **<0.001** | |
|  | 1 Presence | 0.02 | 0.12 | 0.01 | 0.19 | 0.853 | |
|  | 2 Proprioception | 0.78 | 0.14 | 0.50 | 5.53 | **<0.001** | |
|  | 3 Disturbance | -0.44 | 0.15 | -0.29 | -3.01 | **0.003** | |
|  | 4 Interoception | 0.25 | 0.12 | 0.16 | 2.02 | **0.045** | |
| *Note.* *B* = unstandardized regression coefficient; *SE_β_* = standard error of the coefficient; *β* = standardized coefficient. Bold values indicate significant *p-*values at *p* < .05. | | | | | | |  |

**Supplementary Table 4.** Summary of analysis assessing the moderation of the MAIA-2 score on the relationship between the four communities and art appraisals (*Liking*, *Meaning*, *Interest*)

| Appraisal | **Liking** | | | **Meaning** | | | | **Interest** | | |  |  |
| --- | --- | --- | --- | --- | --- | --- | --- | --- | --- | --- | --- | --- |
| *Predictors* | *B* | *t* | *p* | | *B* | *t* | *p* | | *B* | *t* | | *p* |
| *Intercept* | 4.50 | 0.11 | **<0.001** | | 2.58 | 2.58 | **<0.001** | | 3.11 | 3.51 | | **0.001** |
| Community 1 *Presence* | 0.90 | 3.56 | **<0.001** | | 1.21 | 1.21 | **0.038** | | 0.26 | 0.34 | | 0.737 |
| MAIA | 0.45 | 2.95 | 0.003 | | 0.60 | 0.60 | **0.002** | | 0.37 | 1.43 | | 0.154 |
| Community 1 *Presence* * MAIA | -0.18 | -1.37 | 0.171 | | -0.26 | -0.26 | 0.123 | | 0.06 | 0.25 | | 0.803 |
| *Intercept* | 4.61 | 9.10 | **<0.001** | | 2.68 | 4.24 | **<0.001** | | 3.25 | 3.77 | | **<0.001** |
| Community 2 *Proprioception* | 0.90 | 1.80 | 0.073 | | 0.84 | 1.36 | 0.176 | | -0.03 | -0.04 | | 0.969 |
| MAIA | 0.42 | 2.84 | **0.005** | | 0.57 | 3.10 | **0.002** | | 0.32 | 1.30 | | 0.196 |
| Community 2 *Proprioception* * MAIA | -0.14 | -0.97 | 0.334 | | -0.10 | -0.55 | 0.584 | | 0.19 | 0.80 | | 0.426 |
| *Intercept* | 4.52 | 8.43 | **<0.001** | | 2.46 | 3.67 | **<0.001** | | 2.79 | 3.04 | | **0.003** |
| Community 3 *Disturbance* | -0.79 | -1.59 | 0.113 | | 0.13 | 0.20 | 0.839 | | 0.65 | 0.76 | | 0.448 |
| MAIA | 0.45 | 2.87 | **0.005** | | 0.63 | 3.25 | **0.001** | | 0.46 | 1.73 | | 0.084 |
| Community 3 *Disturbance* * MAIA | 0.17 | 1.19 | 0.233 | | -0.10 | -0.57 | 0.568 | | -0.18 | -0.72 | | 0.470 |
| *Intercept* | 4.29 | 8.04 | **<0.001** | | 2.26 | 3.38 | **0.001** | | 2.76 | 3.11 | | **0.002** |
| Community 4 *Interoception* | -0.81 | -1.63 | 0.103 | | 0.28 | 0.44 | 0.659 | | 0.58 | 0.71 | | 0.480 |
| MAIA | 0.51 | 3.28 | **0.001** | | 0.69 | 3.57 | **<0.001** | | 0.47 | 1.83 | | 0.068 |
| Community 4 *Interoception* * MAIA | 0.21 | 1.48 | 0.141 | | -0.09 | -0.48 | 0.632 | | -0.06 | -0.24 | | 0.807 |
| *Note.* *B* = unstandardized regression coefficient. Bold values indicate significant *p-*values at *p* < .05. | | | | | | | | | | | | |

# Supplementary Information and Analyses

## Exploratory Analyses

### Impact Fear of Height on Art/Bodily Experience

To control whether fear of height impacted art experience, we ran a series of linear regressions with fear of height as a predictor and the art experiences as the outcome. Fear of height did not predict *Wonder*, *Liking*, *Meaning*, *Interest, Self-reflection, Perspective Change, and Transformation* (all *p*_s_ < Bonferroni corrected alpha: 0.05/9 = .005). Fear of heights predicted *Awe*, F(1, 233) = 8.64, p = .004, and overall enjoyment of the experience, F(1, 223) = 27.37, p < .001, suggesting that people who had more fear of height, reported higher awe and did not enjoy their bodily experience.

## Network Estimation

Network estimation via the TMFG algorithm begins by connecting the four terms in a tetrahedron that have the highest sum of zero-order correlations with all other terms. Then, the algorithm connects the next term with the largest sum of correlations to three nodes already in the network. This process iteratively continues until every term has been added to the network.

## Structural Consistency of EGA Analysis

## Although the EGA algorithm is deterministic, assessing how stable and generalizable the results are essential. Sample-specific aspects (e.g., sample measures, sample size) may lead to dimensional structures that may not generalize to other samples, potentially leading to inconsistencies in the number and content of communities. Thus, we controlled for structural consistency using bootstrapping and confirmatory factor analysis.

### Bootstrapping

#### To address the reproducibility of psychometric networks and overcome limitations in generalizability, Bootstrap Exploratory Graph Analysis has been introduced (bootEGA; Christensen & Golino, 2021). BootEGA allows the researcher to estimate and evaluate the stabilities of dimensions identified by EGA. We employed 1000 bootstrap samples using bootEGA in *R*. Descriptives showed that the four dimensions are relatively stable (median, i.e., the median value of each correlation between the terms in the network = 4, SD = 0.55, and 95% CI [2.94, 5.06]). The likelihood of the dimensions showed that the four communities were replicated most of the times in comparison to a dimensional structure with one, two or three communities (673 times; 67%). This analysis confirmed that four communities were the most stable dimensional organization of the data.

The item stability across all bootstrap samples is displayed in **Supplementary Figure 2**, which indicates the community to which item belonged to as well as the proportion of times the clustering of each item with the *same* dimension across the replica. Items with lower stability were “existence” (27%), “watched” (55%), “exhausted “(57%), and “grounded” (66%). These items were clustered with different communities across the different bootstrap samples (cross-loadings). The low item consistency of “existence” (27%) explains why this dimension was not stable between EGA and bootEGA. Items that achieved the value 100% or close to 100% stability were “body,” “posture,” “balance,” and “movement”.

It has furthermore been suggested to compare the original (EGA) and median (bootEGA) network results (Christensen & Golino, 2021). We found that “existence” grouped in Community 1 in bootEGA, but in community 2 in EGA (see **Supplementary Figure 2 and 3**).

### Confirmatory Factor Analysis

Because EGA and bootEGA are exploratory, we also applied further confirmatory techniques to estimate how well the data fit this structure (Kan et al., 2019). Confirmatory Factor Analysis (CFA) with the dimensions specified by EGA was performed to provide evidence for the fit of the four-dimensional structure of the body measurement. The results indicated an acceptable absolute fit of the four dimensions to the model [𝜒^2^=530.21, *df*=344.000, 𝜒^2^/*df*=1.54, CFI=0.935, TLI=0.928, NFI=0.835, NNFI=0.928, RMSEA=0.048, SRMR, 0.078] (see **Supplementary Figure 4** for CFA plot).
